# A Critical Compilation of Energy Levels, Spectral Lines, and Transition Probabilities of Singly Ionized Silver, Ag II

**DOI:** 10.6028/jres.118.009

**Published:** 2013-04-15

**Authors:** Alexander Kramida

**Affiliations:** National Institute of Standards and Technology, Gaithersburg, MD 20899

**Keywords:** atomic energy levels, critically evaluated data, ionization limit, singly ionized silver, transition probabilities, wavelengths

## Abstract

All available experimental measurements of the spectrum of the Ag^+^ ion are critically reviewed. Systematic shifts are removed from the measured wavelengths. The compiled list of critically evaluated wavelengths is used to derive a comprehensive list of energy levels with well-defined uncertainties. Eigenvector compositions and level designations are found in two alternate coupling schemes. Some of the older work is found to be incorrect. A revised value of the ionization energy, 173283(7) cm^−1^, equivalent to 21.4844(8) eV, is derived from the new energy levels. A set of critically evaluated transition probabilities is given.

## 1. Introduction

Ag II, like the isoelectronic neutral palladium, has the ground term 4d^10 1^S_0_. Promotion of one electron from the 4d shell produces a relatively simple system of level series 4d^9^*nl*. Promotion of two electrons results in complex configurations 4d^8^*nln*′*l*′ with three open shells. Two of them, 4d^8^5s^2^ and 4d^8^5s5p, appear below the ionization limit and contribute to the complexity of the observed spectrum.

The most complete previous analysis of the Ag II spectrum was made by Kalus *et al.* [1]. These authors measured the spectrum emitted by a pulsed hollow-cathode discharge in the region 940 Å to 8500 Å. A good review of the previous work can be found in that paper.

Kalus *et al.* [1] noted that their line list does not include many of the lines reported by earlier observers. In particular, they mentioned that Gilbert [2] was able to observe many more combinations between highly excited levels by using a condensed hollow-cathode discharge. Rasmussen [3,4] reported 40 lines originating from the 4d^9^6p, 4d^8^5s^2^, and 4d^8^5s5p configurations that were not observed by Kalus *et al.* Benschop *et al.* [5] listed 65 lines ascribed to Ag II, 43 of which they classified as transitions involving the highly excited 4d^9^8s and 4d^9^9s configurations. They used two different types of spark discharges as light sources, a sliding spark and a three-electrode vacuum spark. Kalus *et al.* [1] suggested the use of these spark sources as the reason for the appearance of lines from highly excited levels in the spectra observed by Benschop *et al.* [5]. However, other authors who used spark discharges [6,7] have identified lines from only moderately excited configurations (4d^9^5s, 4d^9^6s, 4d^9^5p, 4d^9^5d). The latter two works contain 32 lines not observed by Kalus *et al.*

Benschop *et al.* [5] based their line identifications and new level values on their own measurements and those previously made by Shenstone [6] and Gilbert [2]. Neither of these measurements was especially precise. For example, for strong unblended lines, uncertainties of wave numbers measured by Benschop *et al.* [5] were 0.13 cm^−1^ to 0.5 cm^−1^. Uncertainties in the line lists of Shenstone [6] and Gilbert [2] were in the ranges 0.5 cm^−1^ to 0.9 cm^−1^ and 0.9 cm^−1^ to 3 cm^−1^, respectively. Since the measurements of Kalus *et al.* [1] provided level values with relative uncertainties as small as 0.003 cm^−1^, the line assignments of Benschop *et al.* can now be evaluated much more accurately. Their new level values could possibly be improved by using their measurements together with the new high-precision data. If such an improvement were achieved, the accuracy of the ionization limit derived by Benschop *et al.* from their level values could also be improved.

As mentioned by Kalus *et al.* [1], the listing of only small sets of newly identified lines impedes comparison of observed intensities and makes it difficult to search for Ag II lines in spectra of composite materials. In addition, the very different excitation conditions used make relative intensities reported by different authors incompatible with each other.

Kalus *et al.* [1] presented a good description of all energy levels derived from their measurements, including some revised level designations and eigenvector percentage compositions in the *LS* coupling scheme. However, the new designations of the 4d^9^*nl* levels are hardly better than the previous ones, because the levels are highly mixed in this coupling scheme. As found by Engleman *et al.* [8], these levels in Pd-like spectra are much better described in *JK* coupling.

As noted by Kalus *et al.* [1], several earlier papers gave theoretical and experimental transition probabilities and radiative lifetimes for Ag II. However, the current Ag II line list in the NIST Atomic Spectra Database (ASD) [9] has no transition probability values. Another source of reference data, the Handbook of Basic Atomic Spectroscopic Data [10] lists only provisional theoretical data from Biémont *et al.* [11] without evaluated uncertainties.

The aims of the present paper are: 1) compile a critically evaluated comprehensive list of all observed spectral lines of Ag II with intensities normalized to a common scale; 2) based on the complete line list, re-optimize the energy levels and determine precise Ritz wavelengths for all observed lines; 3) give a good theoretical description of all known energy levels in the most appropriate coupling scheme providing unambiguous level designations; 4) validate the line classifications given by Benschop *et al.* [5] and derive an improved value for the ionization limit; and 5) compile a set of critically evaluated transition probabilities.

## 2. Observed Lines of Ag II

Table 1 is the total list of observed lines of Ag II compiled from the measurements of Kalus *et al.* [1], Rasmussen [3,4], Gilbert [2], Blair [7], and Shenstone [6]. These measurements and their uncertainties are discussed in detail below. The observations of Benschop *et al.* [5] are also discussed. However, since their results were found to be incorrect, they were not included in the new level optimization.

The observed wavelengths and their uncertainties, as given in Table 1, are discussed below in this section. All uncertainties given in this work are meant to be on the level of one standard deviation. The observed relative intensities given in Table 1 were converted to an arbitrary uniform scale as described in Sec. 5.

The Ritz wavelengths given in Table 1 were obtained from the optimized energy levels (see Sec. 3). Both observed and Ritz wavelengths above 2000 Å are given in standard air. They were converted from the vacuum wave numbers using the five-parameter formula of Peck and Reeder [12].

### 2.1 Observations of Kalus *et al.* [1]

As mentioned in the Introduction, the light source used in Kalus et al. [1] was a pulsed hollow cathode discharge. Argon, neon, and helium mixtures were used as carrier gases. Neon and helium were used as it was found by many authors that collisional transfer of charge and energy from He^+^ and Ne^+^ greatly enhances lines from highly excited levels of Ag^+^. Two different measurement techniques were used in two wavelength regions. Measurements of 97 lines in the vacuum ultraviolet (VUV) region below 1800 Å were made on photographic recordings of spectra obtained with a 10.7 m vacuum grating spectrograph. Above 1800 Å, the spectrum was recorded with a Fourier transform spectrometer (FTS); 216 lines were measured in this spectral region.

The wavelength scale of the FTS measurements was calibrated by means of Ar II lines measured by Norlén [13]. Uncertainties of the FTS lines, including the calibration uncertainty, ranged from 0.003 cm^−1^ for strong lines to 0.03 cm^−1^ for the weakest lines.

For the VUV spectrum, the wavelength scale was initially calibrated using internal standards consisting of Ne II lines from the carrier gas and C I, N II, and O II impurity lines, as well as external Cu II standards provided by an auxiliary Cu-Ne hollow cathode. The final calibration included a large number of Ag II Ritz wavelengths of 5s-6p lines, derived from the energy levels established from the FTS measurements. Uncertainties of the VUV lines were in the range from 0.0005 Å for strong lines to 0.002 Å for weak lines.

Nave and Sansonetti [14] have found that the wave number scale of Norlén [13] has a calibration error, and all wave numbers from his paper have to be increased by 6.7 parts in 10^8^. Since all measurements of Kalus *et al.* [1] were ultimately based on the scale of Norlén, I applied the above correction factor to all their wave numbers. The correction amounted to 0.0008 cm^−1^ to 0.0035 cm^−1^ for the FTS measurements and 0.004 cm^−1^ to 0.007 cm^−1^ for the VUV grating measurements. While for the VUV region the correction is well below the stated measurement uncertainties, (7–12)×10^−5^ Å for wavelengths, the correction of the FTS measurements is significant, especially for short wavelengths.

To estimate the wave number uncertainties, I assumed that the measurement uncertainties *δσ* are entirely due to statistical and systematic uncertainties (*δσ*_stat_ and *δσ*_syst_, respectively) in the wave number measurements of symmetric well-resolved features (see, for example, Kramida and Nave [15]):

(1) $$\delta \sigma \approx {\left({\delta \sigma_{\text{stat}}}^{2}+{\delta \sigma_{\text{syst}}}^{2}\right)}^{1/2},$$

(2) $$\delta \sigma_{\text{stat}}\approx W/\left(2S/N\right),$$

where *W* is the full width at half-maximum. The signal-to-noise ratios *S/N* are given in the line list of Kalus *et al.* [1] in the column of line intensities. The values of *W* and *δσ*_syst_ can be estimated as 0.03 cm^−1^ and 0.003 cm^−1^, respectively, from the statement above about the uncertainties of the FTS measurements quoted from [1]. Uncertainties of the VUV grating measurements given in Table 1 were estimated using similar considerations.

To verify the assigned uncertainties, I calculated the energy levels with the least-squares level optimization code LOPT [16] using only the uncorrected wave numbers listed by Kalus *et al.* [1] with the uncertainties assigned as described above. The resulting energy level values were in close agreement with those given by Kalus *et al.*, which confirms that the assigned uncertainties are close to those used by Kalus *et al.* in their level optimization.

In Table 1, the observed wavelengths referred to Kalus *et al.* [1] were obtained from the wave numbers, corrected as described above.

### 2.2 Observations of Rasmussen [3,4]

Rasmussen observed the Ag II spectrum between 2530 Å and 9053 Å with a hollow cathode of pure silver in neon [3,4] and helium [4] discharges. The first paper [3] is a preliminary report of the more extensive studies reported in [4].

**Fig. 1:**
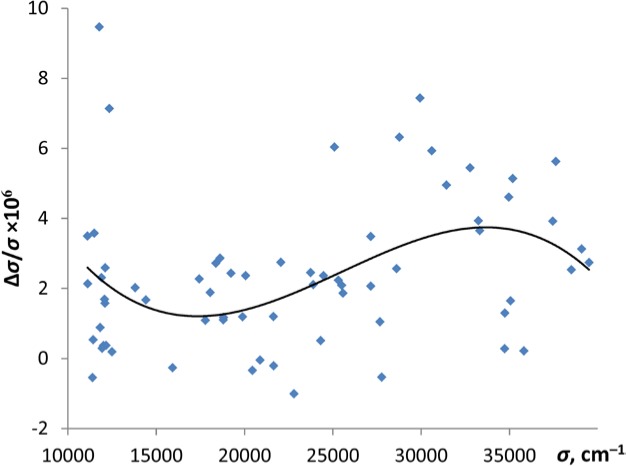
Relative deviations of wave numbers measured by Rasmussen [4] from Ritz wave numbers based on measurements of Kalus *et al.* [1]. The smooth curve is a cubic polynomial that fits the data points.

Rasmussen specified the uncertainty of his wavelength measurements to be about 0.01 Å. He listed the wavelengths rounded off to two places after the decimal point. However, his listed wave numbers are given with higher precision. Comparison of these wave numbers with the Ritz values based mainly on the FTS measurements of Kalus *et al.* [1] reveals that they have a relative statistical uncertainty Δ*σ*/*σ* = 2.0×10^−6^ and a systematic shift smoothly varying from +1 part in 10^6^ at 18000 cm^−1^ to +4 parts in 10^6^ at 33000 cm^−1^ (see Fig. 1).

I removed this systematic shift by subtracting the values represented by the smooth curve shown in Fig. 1 from the wave numbers of Rasmussen. The statistical uncertainties of the resulting wavelengths vary from ±0.005 Å at 2530 Å to ±0.018 Å at 9000 Å.

### 2.3 Observations of Gilbert [2]

In the work of Gilbert [2], the spectrum was excited in a pulsed hollow cathode discharge with a low-pressure helium carrier gas. Enhancement of lines originating from highly excited levels was achieved by using a spark gap in series with the hollow cathode. Wavelength measurements were made in the region from 728 Å to 2600 Å with a 1.5-meter grating vacuum spectrograph and in the region from 4000 Å to 11000 Å with a 3-prism spectrograph. Impurity lines of oxygen, nitrogen, carbon, helium, and hydrogen, as well as Ag II lines previously measured by Shenstone [6] were used as internal standards in the region below 2600 Å. The measurements were believed to be accurate to 0.05 Å in this region (1.4 cm^−1^ at 1900 Å to 5 cm^−1^ at 1000 Å). In the region above 4000 Å, the standards were furnished by an iron comparison spectrum. The measurement uncertainty was estimated as ±1 cm^−1^ over this region (0.16 Å at 4100 Å and 1.0 Å at 9000 Å).

As in the case of the data of Rasmussen, the measurements of Kalus *et al.* [1] now provide an excellent set of internal references. Re-calibration of the wave numbers reported by Gilbert against the Ritz wave numbers derived from the measurements of Kalus *et al.* results in significant improvement of accuracy. The corrected wave numbers and wavelengths obtained from them are given in Table 1. For unblended lines with large or medium intensity, uncertainties are 2 cm^−1^ below 1000 Å (0.011 Å at 728 Å to 0.02 Å at 1000 Å), 1.0 cm^−1^ in the region 1000 Å to 1900 Å (0.010 Å at 1000 Å to 0.03 Å at 1900 Å), 0.5 cm^−1^ in the region 1937 Å to 2186 Å (about 0.02 Å), 0.2 cm^−1^ in the region 4313 Å to 4531 Å (about 0.04 Å), and 0.4 cm^−1^ above 6279 Å (0.2 Å at 6279 Å to 0.3 Å at 9000 Å). For weak or unresolved lines, uncertainties are much greater, about 3 cm^−1^ (0.04 Å at 1080 Å and 2.3 Å at 9000 Å). The correction applied to wave numbers measured by Gilbert [2] varies between −3.4 cm^−1^ and +2.3 cm^−1^, being the greatest at 1370 Å.

Three lines listed by Gilbert with wave numbers 94429.6 cm^−1^, 89847.3 cm^−1^, and 21499.7 cm^−1^ were classified as transitions to or from the odd-parity level designated by Gilbert as 3_2_, located at 133589.4 cm^−1^. Since my energy-level calculations (see Sec. 4) show that this level is not real, and since these three lines cannot be classified as transitions between any known levels, I discarded the spurious level and the lines.

### 2.4 Observations of Blair [7]

Blair [7] photographed the Ag II spectrum in the region 2000 Å to 3400 Å emitted by a hollow cathode. Only 20 newly classified lines were given in this paper. Most of them were remeasured by Kalus *et al.* [1] with much greater accuracy. However, seven lines were not reported elsewhere. To determine their uncertainties, I compared wavelengths reported by Blair with the Ritz wavelengths derived from the measurements of Kalus *et al.* [1]. From this comparison, it follows that the measurements of Blair [7] have a statistical uncertainty of 0.04 Å with a small systematic shift of about +0.01 Å. Since this shift is much smaller than the statistical uncertainty, and all the levels involved are precisely determined by the measurements of Kalus *et al.* [1], I did not remove this shift from the wavelengths reported by Blair.

### 2.5 The Work of Shenstone [6]

All observations reported in the work of Shenstone [6] were made with a spark source. For wavelengths above 2246 Å, Shenstone listed measurements of Exner and Haschek [17], with few exceptions where he gave his own measured values or those from Frings [18]. Most of the lines reported by Shenstone were accurately re-measured by Kalus *et al.* [1]. However, there are 12 lines not reported by other observers, 10 quoted from [17] and two measured by Shenstone. These lines are between 2111 Å and 3270 Å. As in the previous cases, it was possible to re-calibrate the wavelength scales of Shenstone and of Exner and Haschek by using Ritz wavelengths based on the measurements of Kalus *et al.* [1] as internal standards. This procedure revealed the presence of large systematic shifts, which are nearly the same in the measurements of Shenstone and of Exner and Haschek and amount to +0.04 Å at 2111 Å and +0.25 Å at 3270 Å. After removing this shift, the resulting wavelengths reported in Table 1 have statistical uncertainties ranging from 0.03 Å at 2111 Å to 0.05 Å at 3270 Å. There is one exception for the weak unresolved line at 2829.0 Å, which deviates from the (much more accurate) Ritz wavelength by 0.4 Å (5 cm^−1^). For this line, I adopted the uncertainty of 0.4 Å.

### 2.6 The Work of Benschop *et al.* [5]

Benschop et al. [5] observed the VUV spectrum of singly and multiply ionized silver in the region 500 Å to 2200 Å by using two light sources: 1) a sliding spark with a ceramic or quartz spacer, and 2) a triggered vacuum spark. Three high-resolution vacuum grating spectrographs facilitated wavelength measurements with uncertainties about or below 0.005 Å. Cu II lines excited in a copper hollow cathode lamp in a helium atmosphere were used as standards in the region above 700 Å. Between 500 Å and 700 Å, either O, C, and N lines or some silver lines measured in the higher orders were used as internal standards. The total number of observed spectral lines was over 10000. Most of these lines were found to be due to Ag III–V [19,20]. Separation of lines belonging to different ionization stages was achieved by varying the operational conditions of the discharges.

Benschop *et al.* [5] stated that they re-measured many of the previously known Ag II lines with greater accuracy and confirmed the previous analysis. However, they listed only 64 lines that were newly classified in their work and gave revised values of 74 energy levels. Thirty-two of the new lines were classified as transitions between levels that are now known with high accuracy from Refs. [1,3-4]. Only one of these lines (1313.809 Å, 4d^9^5p ^3^P°_1_ – 4d^9^8s ^1^D_2_) agrees with the Ritz wavelength (1313.804(4) Å) within the combined uncertainties. The root-mean-square (rms) deviation of the measured wavelengths from the Ritz values is 0.09 Å, i.e., 18 times the claimed measurement uncertainty. The other 32 lines ascribed by Benschop *et al.* to the previously unknown 4d^9^9s configuration and 4d^8^5s^2 1^S_0_ level have similarly large deviations of wavelengths from the Ritz values. These deviations appear to be random and have both large positive and large negative values. They do not show any regular trend that could be interpreted as a smoothly varying calibration error. The rms deviation of the energy level values obtained by Benschop *et al.* from the new, much more accurate values (see Table 2 and Sec. 3) is about 6 cm^−1^, while the uncertainty implied by their wavelength measurements should not exceed 0.5 cm^−1^. The values of 4d^9^6p ^3^P°_2_ and 4d^9^5d ^3^D_2_ deviate from those of Kalus *et al.* by 21 cm^−1^ and –42 cm^−1^, respectively. In addition, the observed relative intensities of the lines have no correlation with calculated radiative rates (see Sec. 6). Therefore, the entire analysis of Benschop *et al.* [5] must be deemed incorrect, and their results must be disregarded, including the identification of the missing ^1^S_0_ level of the 4d^8^5s^2^ configuration, the 4d^9^9s levels, and their derivation of the ionization energy.

A special note should be made about the line at 1160.887 Å assigned to the 4d^10 1^S_0_ − 4d^9^5p ^3^P°_2_ transition [5]. Its wave number does not agree with the energy of the 4d^9^5p ^3^P°_2_ level given by Benschop *et al.* [5]. The closest match is with the 4d^9^5p ^3^P°_0_ level, which differs from the wave number of this line by 5 cm^−1^. Neither classification is feasible, since all 4d^9^5p levels have allowed (electric-dipole) radiative transitions to lower even-parity levels of the 4d^9^5s configuration. Forbidden transitions from these levels must have very small branching ratios.

### 2.7 Other Observations

Several lines of Ag II in the range 2243 Å to 8404 Å were found by Reid *et al.* [21,22] to exhibit lasing in hollow cathode discharges filled with Ne or He. Eleven lasing lines were identified with known Ag II transitions listed in Refs. [1,2]. Several unidentified lines reported in other papers of the same team [23,24] were found by Reid *et al.* [21] to be diffraction-grating ghost lines. The identified lasing lines are marked in Table 1 with a corresponding note.

Tables of the Massachusetts Institute of Technology (MIT) [25] include wavelengths and relative intensities of about 340 lines of Ag I and Ag II in the range 2000 Å to 8274 Å. Most of the lines ascribed to Ag II in these tables have been observed by Kalus *et al.* [1] and Gilbert [2]. The MIT tables should be used with caution, as most Ag II wavelengths referred to MIT measurements, including those given with three places after the decimal point, appear to possess a large systematic shift on the order of 0.2 Å. Because of this shift, I did not make use of these tables.

## 3. Optimized Energy Levels

After the systematic shifts have been removed, and wavelength measurement uncertainties have been assessed, the level optimization is a straightforward procedure. I used the least-squares level optimization code LOPT [16] and included only the measurements of Kalus *et al.* [1], Rasmussen [3,4], Blair [7], and Shenstone [6], which makes a total of 450 lines. The level-optimization problem defined by this line list has 353 degrees of freedom (DF). The resulting ratio of the residual sum of squares (RSS) to the number of degrees of freedom RSS/DF = 0.78, which indicates that the wavelength values and their uncertainties are statistically reasonable. The optimized energy levels and their uncertainties are given in Table 2.

As noted by Kalus *et al.* [1], the uncertainty of the absolute level values is determined mainly by three observed spectral lines at 1106, 1112, and 1196 Å connecting the 4d^10 1^S_0_ ground level with the three *J* = 1 levels of 4d^9^5p (^3^D°, ^1^P°_1_, and ^3^P°_1_, respectively). They estimated this uncertainty as ±0.1 cm^−1^ and estimated the uncertainties of intervals between the excited levels to be in the range ±0.001 to ±0.01 cm^−1^. The newly optimized energy levels given in Table 2 agree very well with those given by Kalus *et al.* [1]. Excitation energies of all levels are greater than those given by Kalus *et al.* by 0.04 cm^−1^ on average, which is well within the uncertainty ±0.1 cm^−1^ specified by Kalus *et al.* Separations of all excited levels from 4d^9^5p ^3^P°_1_ are all within the range of uncertainties given by Kalus *et al.* The level 4d^9^5p ^3^P°_1_ was chosen as the base for determining the relative uncertainties of excited levels, because it has the greatest number of accurately measured connecting lines. In most cases, the number of significant figures given in the energy value is determined by the value of the relative uncertainty. In some cases, an extra digit was required in the energy value in order to exactly match some of the observed wavelengths.

The only questionable level in Table 2 is 4d^9^6p ^3^P°_0_, which is determined by a single line at 9052.70 Å tentatively classified by Rasmussen [4] as a transition from this level to 4d^9^6s ^3^D_1_. Another potentially strong transition from this level to 4d^9^5s ^3^D_1_ is predicted to occur at 1081.9340(3) Å and could have been masked by the much stronger line at 1082.1458 Å in the spectrum observed by Kalus *et al.* [1].

The four 4d^9^8s levels are determined with uncertainties of about 0.2 cm^−1^ from lines of transitions to 4d^9^5p and 4d^9^6p measured by Gilbert [2].

The Landé factors included in Table 2 are from Moore [26]. Designations of the levels are discussed in Sec. 5.

## 4. Ionization Limit

The previously accepted value of the first ionization limit, 173277.4 cm^−1^, was derived by Benschop *et al.* [5] from the 4d^9^*n*s (*n* = 5–8) ^1,3^D series. They noted that their newly identified (now discarded) 4d^9^9s ^1,3^D levels are “slightly perturbed” and did not include them in the derivation of the limit. Benschop *et al.* did not specify the uncertainty of their value. Since now the 4d^9^*n*s series are known much more accurately, it is possible to derive an improved limit value from the same series using similar quantum-defect expansion formulas. For that I used the computer program RITZPL by Sansonetti [27]. Each of the four four-member series was exactly fitted using the three-constant extended Ritz formula

(1) $$\delta_{n}=c_{0}+c_{1}/{\left(n-\delta_{n}\right)}^{2}+c_{2}/{\left(n-\delta_{n}\right)}^{2}+c_{3}/{\left(n-\delta_{n}\right)}^{6},$$

where *c_i_* are the fitted constants and *δ_n_* is the quantum defect describing an empirical correction to the principal quantum number *n* required for the excitation energy *E_n_* to satisfy the hydrogenic formula:

(2) $$E_{1}-E_{n}=RZ^{2}/{\left(n/\delta_{n}\right)}^{2},$$

where *E*_I_ is the ionization energy, *Z* is the charge of the ionic core, and *R* is the mass-corrected Rydberg constant.

The mean of the four resulting values of the ionization limit is 173283 cm^−1^ with a standard deviation of 7 cm^−1^. In this derivation, I used the known value of the 4d^9 2^D_5/2_–^2^D_3/2_ separation in Ag III, 4609.2 cm^−1^ [20].

To verify that higher-order terms omitted in Eq. (1) do not significantly influence the results, I made a similar derivation of the ionization limit for neutral palladium, where an accurate value of the limit, 67241.3(8) cm^−1^ was derived by Baig *et al.* [28] from high-member absorption series 4d^10 1^S_0_ – 4d^9^*n*p (*n* = 10–30) and *n*f (*n* = 9–17). For comparison with Ag II, only the 4d^9^_5/2_*n*s ^2^[5/2]_3_ series can be used in Pd I, because the *n* = 8 members are not known in the Pd I 4d^9^_3/2_*n*s ^2^[3/2]_1_ and ^2^[3/2]_2_ series, and the 4d^9^_5/2_*n*s ^2^[5/2]_2_ series is strongly perturbed. From the exact fit of the formulas (1) and (2) with the first four members (*n* = 5–8) of the 4d^9^_5/2_*n*s ^2^[5/2]_3_ series precisely measured by Engleman *et al.* [8], I obtained the limit at 67241.0 cm^−1^, differing from the adopted value of Baig *et al.* by only 0.3 cm^−1^. This, as well as the small variation between the limit values obtained from the four different series, confirms the validity of the procedure for Ag II and allows me to adopt the resulting value of 173283(7) cm^−1^ for the Ag II ionization limit.

## 5. Theoretical Interpretation of the Energy Levels

Atomic structure calculations were made in this work with the Cowan code package [29]. The following configuration sets were used: for even parity, [4p^6^]4d^10^, 4d^9^*n*s, 4d^9^*n*d (*n* = 5–9), 4d^9^5g, 4d^8^5s^2^, 4d^8^5p^2^, 4d^8^5d^2^, 4d^8^5s5d, 4d^8^5p*n*f (*n* = 4, 5), 4p^5^4d^10^5p, 4p^5^4d^10^*n*f (*n* = 4, 5); for odd parity, [4p^6^]4d^9^*n*p (*n* = 5–8), 4d^9^*n*f (*n* = 5–7), 4d^8^5s5p, 4d^8^5p5d, 4d^8^4f5s, 4d^8^5d*n*f (*n* = 4, 5). These sets somewhat extend the ones used by Campos *et al.* [30] and found by them to produce transition rates and radiative lifetimes that agree well with experiment and other high-quality calculations. All known energy levels from Table 2, except for the questionable 4d^9^6p ^3^P°_0_, were included in the least squares fitting (LSF) procedure. In LSF, the average energies of the 4d^10^, 4d^9^*n*s (*n* = 5–7), and 4d^9^5p configurations were varied independently, while the average energies of the 4d^9^*n*s (*n* = 8, 9), 4d^9^*n*d (*n* = 5–9), [4d^8^5*l*^2^ (*l* = s, p, d), 4d^8^5p*n*f (*n* = 4, 5)] (even parity), 4d^9^*n*p (*n* = 6–8), 4d^9^*n*f (*n* = 4–8), and [4d^8^5s5p, 4d^8^5p5d, 4d^8^4f5s, 4d^8^5d*n*f (*n* = 4, 5)] (odd parity) configurations were linked together in the corresponding groups with ratios within each group fixed at the Hartree-Fock (HF) values. Thus, the positions of unknown highly excited configurations were scaled according to the experimentally known lowest configuration in each group. Similarly, the values of the *ζ*_4d_ parameter for the 4d^9^*nl* and 4d^8^*nlnl′* configuration groups were linked together in two groups for each parity with ratios fixed at the HF values. Similar linking of the electrostatic parameters *G^k^*(4d, *nl*) and *F^k^*(4d, *nl*) was also enforced along series of configurations. The effective parameter *α*_4d_ was found to be 38.8(3) cm^−1^ from the fitting of the 4d^8^5s^2^ configuration and then the same value was used in all other 4d^8^*nlnl′* configurations of both parities. Very small or poorly defined single-configuration parameters, such as *ζ*_5d_ for 4d^9^5d, as well as all configuration-interaction parameters were fixed at the HF level for *ζ_nl_* and 0.8×HF for all others.

I fitted 43 known levels of even parity with 17 free parameters, with a standard deviation of 23 cm^−1^, and 55 odd-parity levels with 18 free parameters, with a standard deviation of 63 cm^−1^.

In these calculations, I found that the 4d^9^*nl* configurations are best described in the *JK* coupling scheme, in which the purity of levels varies from 79 % for 4d^9^5p to about 95 % for 4d^9^5d and all 4d^9^*nl* with *n* > 5. Therefore, the configuration and term labels, as well as percentage compositions for these configurations are given in Table 2 in this coupling scheme, providing unambiguous physically meaningful designations. For the 4d^8^5s5p configuration, *LS* coupling of the type 4d^8^ + 5s5p was found to give a slightly better purity of levels, 56 %, compared to the coupling 4d^8^5s + 5p, 54 %. For some of the levels of this configuration, the leading percentage is as small as 25 %. In these cases, the configuration and term labels have little physical meaning and are used for bookkeeping purpose only.

## 6. Relative Intensities of Observed Lines

As noted in the Introduction, the relative intensities of lines observed with different light sources and with different registration equipment are vastly different. In order to give a consistent set of relative intensities, they must be converted to the same scale. To account for the different excitation conditions in various light sources, the observed line intensities can be approximated by local thermodynamic equilibrium (LTE) with an effective excitation temperature pertinent to each light source, and then scaled to the same excitation temperature. In reality, the LTE approximation describes the observed intensities only qualitatively, with deviations in both directions up to an order of magnitude. However, this method results in much better qualitative agreement between relative intensities observed by different authors. In addition to the different effective temperatures in the light sources, the observed intensities are strongly affected by different responses of the dispersive elements and detectors to different wavelengths. These variations can also be accounted for and removed. The general method for doing this was described in my recent paper on In II [31]. It relies on radiative transition rates *A_ki_* calculated with Cowan’s codes (see the previous section), and on the LTE relation between these transition rates and the observed intensities *I*_obs_:

(4) $$I_{\text{obs}}\propto \left(g_{k}A_{\mathrm{ki}}/\lambda \right)\exp\left(-E_{k}/kT_{\text{eff}}\right),$$

where *g*_k_ and *E_k_* are the statistical weight and energy of the upper level, *λ* is the central wavelength of the line in vacuum, *k* is the Boltzmann constant, and *T*_eff_ is the effective temperature.

## 7. Transition Probabilities

Experimental transition probabilities (*A*-values) for transitions originating from the 4d^8^5s^2^, 4d^9^6s, and 4d^9^5d configurations have been obtained by Campos *et al.* [30] using a combination of theoretical lifetimes and measured branching fractions. The calculations were made with Cowan’s codes [29] using two models, one with semi-empirical account for core polarization (CP), and the other one without core polarization corrections, but with an extended set of interacting configurations that effectively introduced the core-polarization effects. In both models, the Slater parameters were adjusted by the LSF method to fit experimental energy levels. The CP calculations were deemed to be more accurate and were used to derive the radiative lifetimes. The accuracy of the calculated lifetimes was indirectly confirmed by a close agreement of the lifetimes in the homologous spectrum Cu II, calculated using a similar method, with experiments.

The radiative lifetimes of the 4d^9^5p levels were measured by Biémont *et al.* [11] using selective laser excitation of a fast ion beam. These experimental radiative lifetimes were used in that work to renormalize the calculated transition probabilities. In this procedure, the calculated *A*-values for all transitions originating from a common upper level were multiplied by a factor equal to the ratio of the measured and calculated lifetimes. This factor was equal to 1.06 on average. The calculations were made with Cowan’s codes [29] using an extended set of interacting configurations (17 of even parity and 13 of odd parity) including configurations with an open 4p shell to mimic the core-polarization effects. The calculated eigenvalues were adjusted to the available observed energy levels by a LSF procedure.

Relative *A*-values of the 4d^9^5s-4d^9^5p transitions were determined by Ferrero *et al.* [32] from measurements of emission line intensities in an optically thin laser-produced plasma. They were placed on an absolute scale by normalizing to their own HFR (Hartree-Fock-Relativistic) calculations with Cowan’s codes [29]. Ferrero *et al.* assumed that their HFR calculations are accurate to 5 %. However, these calculations were single-configuration and, as evidenced by more extensive calculations of Biémont *et al.* [11], as well as my own (see Sec. 5), this estimate was far too optimistic. Nevertheless, the experimental results of Ferrero *et al.* are still useful. They did not give the measured intensity values, but it was possible to deduce them from the given *A*-values using the fact that the LTE condition was experimentally confirmed for the studied plasma source. I renormalized these relative intensities to the lifetimes measured by Biémont *et al.* [11]. For some radiative branches that were not observed by Ferrero *et al.* [32], I used calculated *A*-values from Biémont *et al.* [11]. For five weak transitions, where the uncertainties of the measured branching fractions were too large (from the 4d^9^4p ^3^D°_1_ level), or the radiative lifetime was not reliably measured (from the 4d^9^4p ^1^P°_1_ level), I used the Boltzmann equation (4) to deduce the *A*-values.

It should be noted that all three sources discussed above [11,30,32] used the energy levels from Benschop *et al.* [5] in their LSF calculations. Although the analysis of the latter authors was incorrect (see Sec. 2.6), the energy levels given by them deviate from the true values by only a few reciprocal centimeters, which does not introduce noticeable errors in calculations of *A*-values.

**Fig. 2:**
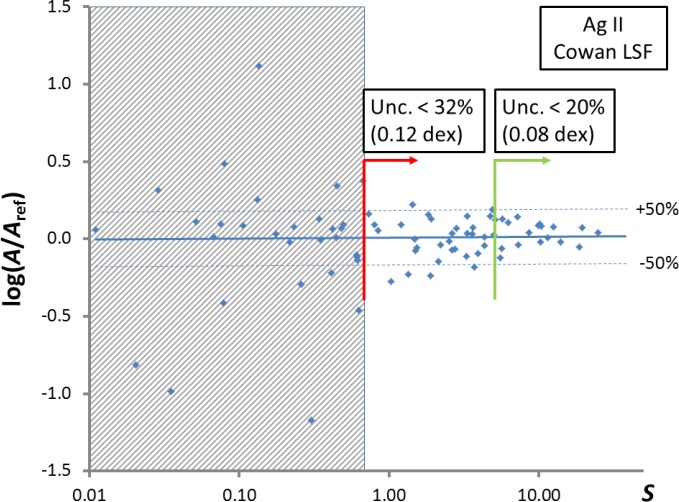
Estimation of uncertainties of calculated *A*-values for Ag II. *S* is the line strength, and the reference values *A*_ref_, having uncertainties smaller than 20 %, are selected from three sources described in the text.

The *A*-values from the above three sources comprised a sufficiently large set of reference data to estimate the uncertainties of my transition-probability calculations described in Sec. 5. The procedure is illustrated in Fig. 2.

For strong lines (line strength *S* > 5.65), the average deviation from the reference *A*-values is about 20 %. For medium-strength lines (0.32 < *S* < 5.65), the average deviation is 32 %. I adopt these average deviations as estimates of uncertainties and extrapolate them to other calculated *A*-values with line strengths from the corresponding ranges.

In the shaded region of Fig. 2 (very weak lines with *S* < 0.32), the calculations are unusable due to large uncertainties (factor of two or greater).

In such comparisons one should use the logarithms of *A*-values or log(*gf*) rather than *A*-values themselves, because of statistical properties of quantum-mechanical calculations. For the quantities log(*A*) or log(*gf*), statistical distribution of deviations from the true values is usually much closer to normal than for the *A*-values, which allows one to use the term “uncertainty” with a well-defined meaning (in the case of the present work, the one-standard-deviation uncertainty of log(*A*) is ±0.08 for strong lines with *S* > 5.65, see Fig. 2).

Together with calculated *A*-values, Cowan’s codes [29] provide values of the cancellation factor CF,

(5) $$\text{CF}=\left(S^{+}+S^{-}\right)/\left(S^{+}+|S^{-}|\right),$$

where *S*^+^ and *S*^−^ are sums of positive and negative contributions to *S*, respectively. Small absolute values of CF indicate that the corresponding *A*-values are unreliable. In the case of my Ag II calculations, a plot similar to Fig. 2, but with CF instead of *S* on the horizontal axis, reveals that for |CF| < 0.1 the calculated *A*-values have uncertainties greater than a factor of 2. Such *A*-values are not included in Table 1.

All adopted *A*-values from the three published sources and the present work are given in Table 1. For the reasons discussed above, uncertainties of the adopted *A*-values are specified in Table 1 with a letter code instead of numerical values. The letter code is explained in Table 3.

In addition to the papers discussed above, there are several other publications involving calculated transition probabilities and measured radiative lifetimes in Ag II. They are not discussed here because their accuracy was found to be lower than for the used sources. A complete list of relevant papers can be retrieved from the NIST Atomic Transition Probability Bibliographic Database [33].

## 8. Conclusions

In the present paper, critically evaluated energy levels, wavelengths, and transition probabilities have been derived for the Ag II spectrum from available experimental and theoretical data. The level list includes 98 excited energy levels having relative uncertainties ranging from 0.0012 cm^−1^ to 0.24 cm^−1^. The uncertainty of the connection between the excited level system and the ground level is 0.06 cm^−1^. The line identifications and level values of the 4d^9^9s configuration from the work of Benschop *et al.* [5] have been found incorrect. A revised value of the first ionization limit, 173283(7) cm^−1^, equivalent to 21.4844(8) eV, has been derived from four 4d^9^*n*s ^1,3^D Rydberg series (*n* ≤ 8). The complete line list includes 452 observed spectral lines between 728 Å and 9053 Å. Transition probabilities have been critically assessed for 237 transitions; 141 of them have been calculated in the present work. Uncertainties of the assessed transition probabilities range from 2 % to 75 %; for 226 transitions, the uncertainties are smaller than 35%.

## Figures and Tables

**Table 1:** Spectral lines of Ag II

*I*_obs_^a^ arb.u.	*λ*_obs_^b^ Å	*σ*_obs_ cm^−1^	*λ*_Ritz_ Å	Δ*λ*_obs−Ritz_ Å	Lower level		Upper level		Line Ref.^c^	*A* s^−1^	Acc.^d^	TP Ref.^e^	Note^f^
1400	9052.697(18)	11043.401			4d^9^_3/2_6s	^2^[3/2]_1_	4d^9^_3/2_6p	^2^[1/2]°_0_	R40bc	4.1e+07	C	TW	S
2900	9001.362(18)	11106.381	9001.360(11)	0.002	4d^9^_5/2_6s	^2^[5/2]_2_	4d^9^_5/2_6p	^2^[7/2]°_3_	R40bc	2.9e+07	C	TW	
550	8995.174(18)	11114.021	8995.181(14)	–0.006	4d^9^_3/2_6s	^2^[3/2]_2_	4d^9^_3/2_6p	^2^[5/2]°_2_	R40bc	1.10e+07	C	TW	
1300	8775.316(18)	11392.472	8775.306(13)	0.010	4d^9^_3/2_6s	^2^[3/2]_1_	4d^9^_3/2_6p	^2^[5/2]°_2_	R40bc	3.0e+07	C	TW	
2800	8748.317(18)	11427.632	8748.307(11)	0.010	4d^9^_3/2_6s	^2^[3/2]_2_	4d^9^_3/2_6p	^2^[1/2]°_1_	R40bc	4.3e+07	C	TW	
260	8725.9(23)	11457	8726.37(7)	–0.5	4d^9^_5/2_6p	^2^[5/2]°_3_	4d^9^_5/2_7s	^2^[5/2]_3_	G35c	1.8e+07	C	TW	
1600	8705.390(17)	11483.982	8705.390(11)	0.000	4d^9^_5/2_6s	^2^[5/2]_3_	4d^9^_5/2_6p	^2^[7/2]°_3_	R40bc	1.17e+07	C	TW	
220	8541.0(22)	11705	8541.87(5)	–0.9	4d^9^_5/2_6p	^2^[5/2]°_2_	4d^9^_5/2_7s	^2^[5/2]_2_	G35c	2.5e+07	C	TW	
390	8540.184(17)	11706.133	8540.196(10)	–0.011	4d^9^_3/2_6s	^2^[3/2]_1_	4d^9^_3/2_6p	^2^[1/2]°_1_	R40bc	1.4e+06	D+	TW	
2600	8492.643(17)	11771.663	8492.661(17)	–0.018	4d^9^_3/2_6s	^2^[3/2]_2_	4d^9^_3/2_6p	^2^[5/2]°_3_	R40bc	4.3e+07	C	TW	
170	8457.546(17)	11820.513	8457.536(5)	0.010	4d^9^_3/2_6s	^2^[3/2]_2_	4d^9^_3/2_6p	^2^[3/2]°_1_	R40bc				
270	8431.5(21)	11857	8431.20(6)	0.3	4d^9^_3/2_6p	^2^[3/2]°_1_	4d^9^_3/2_7s	^2^[3/2]_1_	G35c	2.4e+07	C	TW	
5000	8403.8338(21)	11896.062	8403.8336(21)	0.0001	4d^9^_5/2_6s	^2^[5/2]_3_	4d^9^_5/2_6p	^2^[7/2]°_4_	K02c	4.7e+07	C	TW	Las
2000	8379.578(4)	11930.496	8379.578(4)	0.000	4d^9^_5/2_6s	^2^[5/2]_2_	4d^9^_5/2_6p	^2^[3/2]°_1_	K02c	5.0e+07	C	TW	
260	8362.4(21)	11955	8361.16(6)	1.2	4d^9^_3/2_6p	^2^[3/2]°_1_	4d^9^_3/2_7s	^2^[3/2]_2_	G35c				
2100	8324.708(3)	12009.132	8324.707(3)	0.002	4d^9^_5/2_6s	^2^[5/2]_2_	4d^9^_5/2_6p	^2^[5/2]°_2_	K02c	4.7e+07	C	TW	Las
2100	8297.8(3)	12048.1	8297.32(6)	0.5	4d^9^_5/2_6p	^2^[7/2]°_4_	4d^9^_5/2_7s	^2^[5/2]_3_	G35c	3.4e+07	C	TW	
1100	8287.284(16)	12063.364	8287.277(7)	0.006	4d^9^_3/2_6s	^2^[3/2]_2_	4d^9^_3/2_6p	^2^[3/2]°_2_	R40bc	3.0e+07	C	TW	
950	8262.874(14)	12099.001	8262.875(5)	–0.001	4d^9^_3/2_6s	^2^[3/2]_1_	4d^9^_3/2_6p	^2^[3/2]°_1_	K02c	4.3e+07	C	TW	
1500	8254.777(4)	12110.868	8254.7772(20)	0.000	4d^9^_5/2_6s	^2^[5/2]_2_	4d^9^_5/2_6p	^2^[5/2]°_3_	K02c	1.4e+07	C	TW	Las
270	8224.770(16)	12155.054	8224.757(5)	0.013	4d^9^_5/2_6s	^2^[5/2]_3_	4d^8^(^3^F)5s5p(^3^P°)	^5^G°_4_	R40bc	1.7e+06	D+	TW	
810	8100.253(20)	12341.90	8100.289(7)	–0.036	4d^9^_3/2_6s	^2^[3/2]_1_	4d^9^_3/2_6p	^2^[3/2]°_2_	R40bc	1.3e+07	C	TW	
220bl	8096.3(20)	12348	8095.17(5)	1.1	4d^9^_3/2_6p	^2^[1/2]°_1_	4d^9^_3/2_7s	^2^[3/2]_2_	G35c	1.8e+07	C	TW	
310	8023.0(3)	12460.7	8022.93(6)	0.1	4d^9^_5/2_6p	^2^[7/2]°_3_	4d^9^_5/2_7s	^2^[5/2]_3_	G35c	8.9e+06	C	TW	
2600	8005.1863(19)	12488.467	8005.1861(15)	0.0001	4d^9^_5/2_6s	^2^[5/2]_3_	4d^9^_5/2_6p	^2^[5/2]°_3_	K02c	3.8e+07	C	TW	Las
420	7957.3(3)	12563.6	7957.08(5)	0.2	4d^9^_3/2_6p	^2^[5/2]°_2_	4d^9^_3/2_7s	^2^[3/2]_1_	G35c	3.7e+07	C	TW	
1800	7930.2(3)	12606.5	7930.19(4)	0.0	4d^9^_5/2_6p	^2^[7/2]°_3_	4d^9^_5/2_7s	^2^[5/2]_2_	G35c	3.2e+07	C	TW	
44	7439.5(17)	13438	7442.896(4)	–3.4	4d^9^_5/2_6s	^2^[5/2]_2_	4d^8^(^3^F)5s5p(^3^P°)	^5^G°_3_	G35c				
250	7239.376(16)	13809.54	7239.381(4)	–0.006	4d^9^_5/2_6s	^2^[5/2]_3_	4d^8^(^3^F)5s5p(^3^P°)	^5^G°_3_	R40bc				
77d	7198.5(16)	13888	7198.4980(21)	0.0	4d^9^_5/2_6s	^2^[5/2]_2_	4d^8^(^3^F)5s5p(^3^P°)	^5^D°_1_	G35c				
200	6998.91(20)	14284.0	6999.06(4)	–0.15	4d^8^(^3^F)5s5p(^3^P°)	^5^D°_1_	4d^9^_3/2_7s	^2^[3/2]_1_	G35c				
68	6939.988(10)	14405.274	6939.987(3)	0.000	4d^9^_5/2_5p	^2^[5/2]°_2_	4d^8^5s^2^	^3^F _2_	K02c				
69bl	6893.7(14)	14502	6892.692(4)	1.0	4d^9^_5/2_6s	^2^[5/2]_2_	4d^8^(^3^F)5s5p(^3^P°)	^5^G°_2_	G35c				
130	6717.36(18)	14882.7	6717.800(4)	–0.44	4d^9^_5/2_6s	^2^[5/2]_3_	4d^8^(^3^F)5s5p(^3^P°)	^5^G°_2_	G35c				
81	6697.223(13)	14927.44	6697.2424(9)	–0.019	4d^9^_3/2_5p	^2^[1/2]°_1_	4d^8^5s^2^	^3^P_2_	R40bc				
64	6505.747(13)	15366.78	6505.7672(7)	–0.021	4d^9^_3/2_5p	^2^[3/2]°_1_	4d^8^5s^2^	^3^P_2_	R40bc				
60	6403.6(12)	15612	6405.327(7)	–1.8	4d^9^_5/2_6s	^2^[5/2]_2_	4d^9^_3/2_6p	^2^[5/2]°_2_	G35c				Las
67	6331.275(12)	15790.24	6331.2484(24)	0.027	4d^9^_5/2_5p	^2^[7/2]°_3_	4d^8^5s^2^	^3^F_3_	R40bc				
110	6279.154(12)	15921.31	6279.149(6)	0.005	4d^9^_5/2_6s	^2^[5/2]_2_	4d^9^_3/2_6p	^2^[1/2]°_1_	R40bc				
38	6255.896(12)	15980.50	6255.8897(24)	0.006	4d^9^_5/2_5p	^2^[3/2]°_1_	4d^8^5s^2^	^3^F_2_	R40bc				
36	5734.226(3)	17434.308	5734.2293(20)	–0.003	4d^9^_5/2_5p	^2^[7/2]°_3_	4d^8^5s^2^	^3^F_2_	K02c				
21000	5622.4822(9)	17780.803	5622.4820(10)	0.0003	4d^9^_5/2_5d	^2^[7/2]_4_	4d^9^_5/2_4f	^2^[9/2]°_5_	K02c	1.4e+08	C	TW	
43	5620.974(3)	17785.574	5620.9724(19)	0.002	4d^9^_5/2_5p	^2^[3/2]°_2_	4d^8^5s^2^	^3^F_3_	K02c				
5400*	5610.6066(16)	17818.438	5610.6081(19)	–0.001	4d^9^_5/2_5d	^2^[7/2]_3_	4d^9^_5/2_4f	^2^[5/2]°_2_	K02c				
5400*	5610.6066(16)	17818.438	5610.6070(10)	–0.0004	4d^9^_5/2_5d	^2^[7/2]_4_	4d^9^_5/2_4f	^2^[7/2]°_4_	K02c	4.0e+07	C	TW	
4200	5589.7828(16)	17884.817	5589.7829(9)	0.0000	4d^9^_5/2_5d	^2^[5/2]_2_	4d^9^_5/2_4f	^2^[7/2]°_3_	K02c	6.9e+07	C	TW	
1700	5588.417(3)	17889.189	5588.4183(9)	–0.002	4d^9^_5/2_5d	^2^[7/2]_4_	4d^9^_5/2_4f	^2^[9/2]°_4_	K02c	1.6e+07	C	TW	
2400	5579.6777(22)	17917.207	5579.6782(9)	–0.0005	4d^9^_5/2_5d	^2^[7/2]_3_	4d^9^_5/2_4f	^2^[7/2]°_3_	K02c	3.7e+07	C	TW	
2300	5573.8261(22)	17936.017	5573.8257(9)	0.0004	4d^9^_5/2_5d	^2^[7/2]_3_	4d^9^_5/2_4f	^2^[7/2]°_4_	K02c	3.2e+07	C	TW	
2300	5558.1401(22)	17986.635	5558.1412(12)	–0.0011	4d^9^_5/2_5d	^2^[5/2]_3_	4d^9^_5/2_4f	^2^[3/2]°_2_	K02c	1.9e+07	C	TW	
12000	5551.9265(9)	18006.765	5551.9264(9)	0.0001	4d^9^_5/2_5d	^2^[7/2]_3_	4d^9^_5/2_4f	^2^[9/2]°_4_	K02c	1.07e+08	C	TW	
2700	5543.2124(18)	18035.072	5543.2121(18)	0.0003	4d^9^_5/2_5d	^2^[5/2]_2_	4d^8^(^3^F)5s5p(^3^P°)	^1^F°_3_	K02c	2.1e+07	C	TW	
27	5538.4490(21)	18050.583	5538.4481(6)	0.0009	4d^9^_3/2_5p	^2^[3/2]°_2_	4d^8^5s^2^	^3^P_1_	K02c				
2300	5493.8309(18)	18197.179	5493.8302(9)	0.0007	4d^9^_5/2_5d	^2^[5/2]_3_	4d^9^_5/2_4f	^2^[7/2]°_3_	K02c	2.8e+07	C	TW	
8300	5488.1567(9)	18215.993	5488.1562(7)	0.0004	4d^9^_5/2_5d	^2^[5/2]_3_	4d^9^_5/2_4f	^2^[7/2]°_4_	K02c	7.8e+07	C	TW	
1500	5478.6597(21)	18247.569	5478.6589(12)	0.0009	4d^9^_5/2_5d	^2^[3/2]_2_	4d^9^_5/2_4f	^2^[1/2]°_1_	K02c	5.4e+07	C	TW	
50	5440.9099(9)	18374.172	5440.9104(5)	–0.0006	4d^9^_3/2_5p	^2^[5/2]°_2_	4d^8^5s^2^	^3^P_2_	K02c				
2200	5424.0511(15)	18431.281	5424.0509(12)	0.0003	4d^9^_5/2_5d	^2^[3/2]_2_	4d^9^_5/2_4f	^2^[3/2]°_2_	K02c	7.4e+07	C	TW	
740	5411.936(3)	18472.540	5411.9338(15)	0.002	4d^9^_5/2_5d	^2^[3/2]_1_	4d^9^_5/2_4f	^2^[3/2]°_1_	K02c	8.9e+07	C	TW	
1800	5410.8114(15)	18476.380	5410.8117(15)	–0.0003	4d^9^_5/2_5d	^2^[3/2]_2_	4d^9^_5/2_4f	^2^[3/2]°_1_	K02c	3.6e+06	D+	TW	
15000	5403.1323(9)	18502.639			4d^9^_5/2_5d	^2^[9/2]_4_	4d^9^_5/2_4f	^2^[11/2]°_5_	K02c	1.8e+08	C	TW	S
20000	5400.1037(9)	18513.016			4d^9^_5/2_5d	^2^[9/2]_5_	4d^9^_5/2_4f	^2^[11/2]°_6_	K02c	1.8e+08	C	TW	S
1500	5392.4686(17)	18539.228	5392.4682(17)	0.0004	4d^9^_5/2_5d	^2^[3/2]_1_	4d^9^_5/2_4f	^2^[5/2]°_2_	K02c	7.9e+07	C	TW	
38	5373.8212(12)	18603.559	5373.8219(5)	–0.0007	4d^9^_3/2_5p	^2^[1/2]°_1_	4d^8^5s^2^	^3^P_1_	K02c				
1600	5362.7877(14)	18641.834	5362.7883(9)	–0.0006	4d^9^_5/2_5d	^2^[3/2]_2_	4d^9^_5/2_4f	^2^[7/2]°_3_	K02c	2.2e+07	C	TW	
2900	5340.0267(11)	18721.291	5340.0267(9)	0.0000	4d^9^_5/2_5d	^2^[9/2]_5_	4d^9^_5/2_4f	^2^[9/2]°_5_	K02c	3.4e+07	C	TW	
1300	5332.5043(14)	18747.700	5332.5049(9)	–0.0006	4d^9^_5/2_5d	^2^[9/2]_4_	4d^9^_5/2_4f	^2^[7/2]°_4_	K02c	2.0e+07	C	TW	
21	5319.7018(20)	18792.818	5319.7015(9)	0.0004	4d^9^_3/2_5p	^2^[1/2]°_1_	4d^8^5s^2^	^3^P_0_	K02c				
48	5317.2122(8)	18801.617	5317.2120(5)	0.0003	4d^9^_5/2_5p	^2^[5/2]°_3_	4d^8^5s^2^	^3^P_2_	K02c				
1200	5312.4571(17)	18818.446	5312.4574(8)	–0.0003	4d^9^_5/2_5d	^2^[9/2]_4_	4d^9^_5/2_4f	^2^[9/2]°_4_	K02c	1.6e+07	C	TW	
27	5198.171(8)	19232.18	5198.1783(8)	–0.007	4d^9^_3/2_5p	^2^[3/2]°_1_	4d^8^5s^2^	^3^P _0_	R40ac				
1700	5142.8156(11)	19439.186	5142.8157(11)	–0.0002	4d^9^_5/2_5d	^2^[1/2]_1_	4d^9^_5/2_4f	^2^[1/2]°_1_	K02c	1.3e+08	C	TW	
720	5137.2470(16)	19460.257	5137.2469(16)	0.0001	4d^9^_5/2_5d	^2^[1/2]_1_	4d^8^(^3^F)5s5p(^3^P°)	^1^D°_2_	K02c	1.8e+07	C	TW	
160	5027.3430(8)	19885.677	5027.3432(3)	–0.0002	4d^9^_3/2_5p	^2^[3/2]°_2_	4d^8^5s^2^	^1^D_2_	K02c	3.4e+05	C+	C05	Las
17	4983.2786(12)	20061.513	4983.2786(4)	0.0000	4d^9^_5/2_5p	^2^[5/2]°_2_	4d^8^5s^2^	^3^P_2_	K02c				
18	4891.335(10)	20438.61	4891.3259(4)	0.009	4d^9^_3/2_5p	^2^[1/2]°_1_	4d^8^5s^2^	^1^D_2_	R40ac	1.00e+05	B	C05	
260	4788.3966(7)	20877.981	4788.3966(3)	0.0000	4d^9^_3/2_5p	^2^[3/2]°_1_	4d^8^5s^2^	^1^D_2_	K02c	9.5e+05	C+	C05	Las
56	4705.873(9)	21244.10	4705.879(3)	–0.007	4d^8^5s^2^	^1^D_2_	4d^9^_5/2_6p	^2^[7/2]°_3_	R40bc				
85	4620.4712(6)	21636.754	4620.4711(3)	0.0001	4d^9^_5/2_5p	^2^[3/2]°_1_	4d^8^5s^2^	^3^P_2_	K02c				
170	4620.0358(6)	21638.793	4620.0355(3)	0.0003	4d^9^_3/2_5p	^2^[5/2]°_3_	4d^8^5s^2^	^1^D_2_	K02c	2.1e+05	C	C05	
23	4533.812(8)	22050.31	4533.8084(4)	0.004	4d^9^_3/2_5p	^2^[5/2]°_2_	4d^8^5s^2^	^3^P_1_	R40ac				
62	4530.145(8)	22068.16	4530.1405(10)	0.005	4d^8^5s^2^	^1^D_2_	4d^9^_5/2_6p	^2^[3/2]°_1_	R40bc				
410	4530.39(4)	22066.99	4530.41(4)	–0.02	4d^9^_5/2_6p	^2^[5/2]°_3_	4d^9^_5/2_8s	^2^[5/2]_3_	G35c	7.4e+06	D+	TW	
210	4515.52(4)	22139.65	4515.58(4)	–0.06	4d^9^_5/2_6p	^2^[5/2]°_3_	4d^9^_5/2_8s	^2^[5/2]_2_	G35c	4.1e+06	D+	TW	
84	4514.053(8)	22146.83	4514.0548(8)	–0.002	4d^8^5s^2^	^1^D_2_	4d^9^_5/2_6p	^2^[5/2]°_2_	R40bc				
410	4494.94(4)	22241.02	4494.92(4)	0.01	4d^9^_5/2_6p	^2^[5/2]°_2_	4d^9^_5/2_8s	^2^[5/2]_2_	G35c	1.1e+07	D+	TW	
110	4493.424(8)	22248.50	4493.4135(6)	0.011	4d^8^5s^2^	^1^D_2_	4d^9^_5/2_6p	^2^[5/2]°_3_	R40bc				
290d	4488.58(20)	22272.5	4488.66(5)	–0.08	4d^9^_3/2_6p	^2^[3/2]°_2_	4d^9^_3/2_8s	^2^[3/2]_2_	G35c	6.8e+06	D+	TW	
210	4479.12(4)	22319.57	4479.09(4)	0.03	4d^9^_5/2_6p	^2^[3/2]°_1_	4d^9^_5/2_8s	^2^[5/2]_2_	G35c	6.4e+06	D+	TW	
290	4449.19(4)	22469.67	4449.17(4)	0.03	4d^9^_3/2_6p	^2^[3/2]°_1_	4d^9^_3/2_8s	^2^[3/2]_1_	G35c	1.0e+07	D+	TW	
580	4430.57(4)	22564.14	4430.63(4)	–0.06	4d^9^_3/2_6p	^2^[5/2]°_3_	4d^9^_3/2_8s	^2^[3/2]_2_	G35c	1.4e+07	D+	TW	
830	4412.00(4)	22659.10	4411.96(4)	0.04	4d^9^_5/2_6p	^2^[7/2]°_4_	4d^9^_5/2_8s	^2^[5/2]_3_	G35c	1.4e+07	D+	TW	
79	4385.0519(6)	22798.342	4385.0517(4)	0.0002	4d^9^_3/2_5p	^2^[1/2]°_0_	4d^8^5s^2^	^3^P_1_	K02c				
290	4364.16(4)	22907.47	4364.09(4)	0.07	4d^9^_3/2_6p	^2^[1/2]°_1_	4d^9^_3/2_8s	^2^[3/2]_2_	G35c	6.6e+06	D+	TW	
210	4333.14(4)	23071.47	4333.16(4)	–0.02	4d^9^_5/2_6p	^2^[7/2]°_3_	4d^9^_5/2_8s	^2^[5/2]_3_	G35c	3.3e+06	D+	TW	
630	4319.59(4)	23143.82	4319.59(4)	0.00	4d^9^_5/2_6p	^2^[7/2]°_3_	4d^9^_5/2_8s	^2^[5/2]_2_	G35c	1.2e+07	D+	TW	
290	4313.51(4)	23176.44	4313.54(4)	–0.02	4d^9^_3/2_6p	^2^[5/2]°_2_	4d^9^_3/2_8s	^2^[3/2]_1_	G35c	1.4e+07	D+	TW	
51	4241.574(9)	23569.52	4241.5562(13)	0.017	4d^8^5s^2^	^1^D_2_	4d^8^(^3^F)5s5p(^3^P°)	^5^G°_3_	R40bc				
83	4240.685(9)	23574.46	4240.698(4)	–0.014	4d^8^5s^2^	^1^G_4_	4d^9^_3/2_6p	^2^[5/2]°_3_	R40bc				
75	4215.734(9)	23713.98	4215.7290(11)	0.005	4d^8^5s^2^	^3^P_0_	4d^9^_5/2_6p	^2^[3/2]°_1_	R40bc				
34	4211.5277(9)	23737.666	4211.5267(3)	0.0010	4d^9^_5/2_5p	^2^[5/2]°_2_	4d^8^5s^2^	^3^P_1_	K02c				
250	4185.4749(5)	23885.420	4185.47499(23)	–0.0001	4d^9^_3/2_5p	^2^[5/2]°_2_	4d^8^5s^2^	^1^D_2_	K02c	3.7e+05	C	C05	
53	4111.8880(5)	24312.868	4111.88813(22)	–0.0001	4d^9^_5/2_5p	^2^[5/2]°_3_	4d^8^5s^2^	^1^D_2_	K02c	1.20e+05	B	C05	
470	4085.9155(5)	24467.412	4085.9155(3)	0.0001	4d^9^_3/2_5p	^2^[5/2]°_3_	4d^8^5s^2^	^1^G_4_	K02c	1.12e+06	B+	C05	Las
220	3985.1904(5)	25085.809	3985.1904(3)	0.0000	4d^9^_5/2_5p	^2^[3/2]°_2_	4d^8^5s^2^	^3^P_2_	K02c				
160	3949.4347(5)	25312.915	3949.4350(3)	–0.0002	4d^9^_5/2_5p	^2^[3/2]°_1_	4d^8^5s^2^	^3^P_1_	K02c				
180	3920.1239(5)	25502.176	3920.1238(5)	0.0000	4d^9^_5/2_5p	^2^[3/2]°_1_	4d^8^5s^2^	^3^P_0_	K02c				
140	3909.3032(5)	25572.763	3909.30327(20)	–0.0001	4d^9^_5/2_5p	^2^[5/2]°_2_	4d^8^5s^2^	^1^D_2_	K02c				
70	3736.516(7)	26755.29	3736.5122(20)	0.004	4d^8^5s^2^	^3^P_2_	4d^9^_5/2_6p	^2^[7/2]°_3_	R40bc				
530	3683.3479(4)	27141.487	3683.3481(3)	–0.0002	4d^9^_5/2_5p	^2^[5/2]°_3_	4d^8^5s^2^	^1^G_4_	K02c	9.9e+05	B+	C05	
260	3682.4638(4)	27148.003	3682.46368(18)	0.0001	4d^9^_5/2_5p	^2^[3/2]°_1_	4d^8^5s^2^	^1^D_2_	K02c	9.0e+04	B	C05	
290	3614.5495(13)	27658.077	3614.5504(5)	–0.0009	4d^8^5s^2^	^3^P_2_	4d^9^_5/2_6p	^2^[5/2]°_2_	K02c				
290	3601.3023(13)	27759.813	3601.3035(4)	–0.0012	4d^8^5s^2^	^3^P_2_	4d^9^_5/2_6p	^2^[5/2]°_3_	K02c				
82	3585.836(8)	27879.54	3585.831(4)	0.005	4d^8^5s^2^	^3^P_2_	4d^8^(^3^F)5s5p(^3^P°)	^5^D°_2_	R40bc				
91	3583.966(8)	27894.09	3583.9647(18)	0.001	4d^8^5s^2^	^3^P_1_	4d^9^_3/2_6p	^2^[1/2]°_1_	R40bc				
150	3534.188(7)	28286.96	3534.1851(9)	0.003	4d^8^5s^2^	^3^P_1_	4d^9^_3/2_6p	^2^[3/2]°_1_	R40bc				
500	3495.2849(4)	28601.787	3495.28495(17)	0.0000	4d^9^_5/2_5p	^2^[7/2]°_3_	4d^8^5s^2^	^1^D_2_	K02c	7.4e+05	C+	C05	
450	3475.8183(4)	28761.969	3475.81840(21)	–0.0001	4d^9^_5/2_5p	^2^[3/2]°_2_	4d^8^5s^2^	^3^P_1_	K02c				
150	3437.707(7)	29080.82	3437.7018(8)	0.005	4d^8^5s^2^	^3^P_2_	4d^8^(^3^F)5s5p(^3^P°)	^5^G°_3_	R40bc				
2600	3384.6244(5)	29536.895	3384.6244(5)	0.0000	4d^8^5s^2^	^3^P_2_	4d^8^(^3^F)5s5p(^3^P°)	^5^D°_1_	K02c				
560	3372.1945(5)	29645.765	3372.19464(18)	–0.0002	4d^9^_3/2_5p	^2^[3/2]°_2_	4d^9^_5/2_6s	^2^[5/2]_3_	K02c	1.00e+06	B	C05	
88	3339.878(7)	29932.61	3339.8913(3)	–0.014	4d^9^_5/2_5p	^2^[7/2]°_4_	4d^8^5s^2^	^1^G_4_	R40bc	2.10e+05	B+	C05	
90	3329.7790(10)	30023.387	3329.78173(17)	–0.0027	4d^9^_3/2_5p	^2^[3/2]°_2_	4d^9^_5/2_6s	^2^[5/2]_2_	K02c				
1400	3269.55(5)	30576.4	3269.56087(18)	–0.01	4d^9^_3/2_5p	^2^[1/2]°_1_	4d^9^_5/2_6s	^2^[5/2]_2_	S28c	2.0e+07	D+	TW	
460	3267.3462(3)	30597.058	3267.34612(14)	0.0001	4d^9^_5/2_5p	^2^[3/2]°_2_	4d^8^5s^2^	^1^D_2_	K02c	8.7e+05	B	C05	
3300	3223.2462(3)	31015.668	3223.24625(15)	–0.0001	4d^9^_3/2_5p	^2^[3/2]°_1_	4d^9^_5/2_6s	^2^[5/2]_2_	K02c	6.9e+06	D+	TW	
1300	3183.9060(3)	31398.882	3183.90601(15)	0.0000	4d^9^_3/2_5p	^2^[5/2]°_3_	4d^9^_5/2_6s	^2^[5/2]_3_	K02c	4.0e+06	B	C05	
1500	3180.7128(3)	31430.403	3180.71265(19)	0.0001	4d^9^_5/2_5p	^2^[7/2]°_3_	4d^8^5s^2^	^1^G_4_	K02c	4.30e+06	B+	C05	Las
97	3146.0704(8)	31776.480	3146.07035(15)	0.0001	4d^9^_3/2_5p	^2^[5/2]°_3_	4d^9^_5/2_6s	^2^[5/2]_2_	K02c				
370	3127.698(6)	31963.13	3127.6972(7)	0.001	4d^8^5s^2^	^3^P_2_	4d^9^_3/2_6p	^2^[3/2]°_1_	R40bc				
100	3051.3341(19)	32763.021	3051.3340(19)	0.0001	4d^8^5s^2^	^3^F_3_	4d^8^(^3^F)5s5p(^3^P°)	^5^D°_3_	K02c				
160	3007.9395(18)	33235.664	3007.9389(6)	0.0005	4d^8^5s^2^	^3^F_2_	4d^9^_5/2_6p	^2^[3/2]°_1_	K02c				
510	3000.8383(6)	33314.309	3000.8384(5)	–0.0001	4d^8^5s^2^	^3^F_2_	4d^9^_5/2_6p	^2^[5/2]°_2_	K02c				
24	2981.018(6)	33535.80	2981.016(3)	0.002	4d^8^5s^2^	^3^F_2_	4d^8^(^3^F)5s5p(^3^P°)	^5^D°_2_	R40bc				
8200	2938.3197(3)	34023.106	2938.31975(12)	0.0000	4d^9^_3/2_5p	^2^[5/2]°_2_	4d^9^_5/2_6s	^2^[5/2]_2_	K02c	3.0e+07	D+	TW	
20	2935.518(6)	34055.58	2935.5204(12)	–0.003	4d^8^5s^2^	^3^F_3_	4d^9^_5/2_6p	^2^[7/2]°_3_	R40bc				
28000	2934.0208(3)	34072.955	2934.02078(13)	0.0000	4d^9^_5/2_5p	^2^[5/2]°_3_	4d^9^_5/2_6s	^2^[5/2]_3_	K02c	7.2e+07	B	C05	
1500	2933.3102(3)	34081.208	2933.3102(3)	0.0000	4d^8^5s^2^	^3^F_4_	4d^8^(^3^F)5s5p(^3^P°)	^5^D°_4_	K02c				
10000	2929.3399(3)	34127.398	2929.33993(12)	0.0000	4d^9^_3/2_5s	^2^[3/2]_2_	4d^9^_5/2_5p	^2^[3/2]°_2_	K02c	2.9e+06	C	F95c	
8400	2919.8283(3)	34238.567	2919.82822(13)	0.0000	4d^9^_3/2_5p	^2^[3/2]°_2_	4d^9^_3/2_6s	^2^[3/2]_1_	K02c	6.0e+07	C+	C05	
14000	2901.8607(3)	34450.553	2901.86068(12)	0.0001	4d^9^_5/2_5p	^2^[5/2]°_3_	4d^9^_5/2_6s	^2^[5/2]_2_	K02c	4.3e+07	D+	TW	
12	2900.428(6)	34467.57	2900.4230(5)	0.005	4d^8^5s^2^	^3^F_3_	4d^9^_5/2_6p	^2^[7/2]°_4_	R40bc				
16000	2896.2710(3)	34517.039	2896.27093(12)	0.0000	4d^9^_3/2_5p	^2^[3/2]°_2_	4d^9^_3/2_6s	^2^[3/2]_2_	K02c	8.4e+07	B	C05	
760	2882.00(5)	34688.0	2882.0780(3)	–0.08	4d^9^_3/2_5p	^2^[3/2]°_2_	4d^9^_5/2_5d	^2^[1/2]_1_	S28c				
9800	2878.78902(25)	34726.640	2878.78902(25)	0.00000	4d^8^5s^2^	^3^F_3_	4d^8^(^3^F)5s5p(^3^P°)	^5^G°_4_	K02c				
3800	2877.92171(25)	34737.105	2877.9218(3)	–0.00008	4d^8^5s^2^	^3^F_2_	4d^8^(^3^F)5s5p(^3^P°)	^5^G°_3_	K02c				
11400	2873.41877(25)	34791.539	2873.41884(13)	–0.00007	4d^9^_3/2_5p	^2^[1/2]°_1_	4d^9^_3/2_6s	^2^[3/2]_1_	K02c	1.06e+08	C+	C05	
550	2859.7113(7)	34958.297	2859.7111(5)	0.0002	4d^8^5s^2^	^3^F_3_	4d^9^_5/2_6p	^2^[5/2]°_2_	K02c				
410	2851.4131(10)	35060.029	2851.4127(5)	0.0004	4d^8^5s^2^	^3^F_3_	4d^9^_5/2_6p	^2^[5/2]°_3_	K02c				
			2850.60148(13)		4d^9^_3/2_5p	^2^[1/2]°_1_	4d^9^_3/2_6s	^2^[3/2]_2_		1.6e+07	D+	TW	P
180	2841.7040(24)	35179.81	2841.7040(20)	0.0001	4d^8^5s^2^	^3^F_3_	4d^8^(^3^F)5s5p(^3^P°)	^5^D°_2_	K02c				
25	2840.638(6)	35193.01	2840.6282(6)	0.010	4d^8^5s^2^	^3^F_2_	4d^8^(^3^F)5s5p(^3^P°)	^5^D°_1_	R40bc				
670	2837.57(5)	35231.1	2837.58520(13)	–0.02	4d^9^_3/2_5p	^2^[3/2]°_1_	4d^9^_3/2_6s	^2^[3/2]_1_	S28c	4.0e+06	B	C05	
740	2829.0(4)	35338	2829.39497(13)	–0.4	4d^9^_5/2_5p	^2^[5/2]°_2_	4d^9^_5/2_6s	^2^[5/2]_3_	S28c				
13000	2815.33114(24)	35509.344	2815.33114(11)	0.00001	4d^9^_3/2_5p	^2^[3/2]°_1_	4d^9^_3/2_6s	^2^[3/2]_2_	K02c	7.1e+07	B+	C05	
400	2801.93(5)	35679.2	2801.91850(24)	0.01	4d^9^_3/2_5p	^2^[3/2]°_1_	4d^9^_5/2_5d	^2^[1/2]_1_	B30				
20000	2799.47584(24)	35710.447	2799.47573(11)	0.00012	4d^9^_5/2_5p	^2^[5/2]°_2_	4d^9^_5/2_6s	^2^[5/2]_2_	K02c	1.0e+08	D+	TW	
900	2791.7477(8)	35809.296	2791.7480(6)	–0.0003	4d^8^5s^2^	^3^F_2_	4d^8^(^3^F)5s5p(^3^P°)	^5^G°_2_	K02c				
1500	2786.31(5)	35879.2	2786.35281(15)	–0.04	4d^9^_3/2_5p	^2^[3/2]°_2_	4d^9^_5/2_5d	^2^[3/2]_2_	S28c				
30000	2767.52713(23)	36122.672	2767.52728(11)	–0.00015	4d^9^_3/2_5s	^2^[3/2]_2_	4d^9^_5/2_5p	^2^[7/2]°_3_	K02c	1.01e+07	B	F95c	
35000	2756.27305(23)	36270.156	2756.27291(11)	0.00014	4d^9^_3/2_5p	^2^[5/2]°_3_	4d^9^_3/2_6s	^2^[3/2]_2_	K02c	2.0e+08	C+	C05	
690	2752.19(5)	36324.0	2752.24198(12)	–0.05	4d^9^_3/2_5p	^2^[3/2]°_2_	4d^9^_5/2_5d	^2^[5/2]_3_	B30	1.0e+06	E	C05	
1290	2743.8981(3)	36433.725	2743.89810(11)	0.0000	4d^9^_3/2_5s	^2^[3/2]_1_	4d^9^_5/2_5p	^2^[3/2]°_2_	K02c	1.1e+06	D+	F95c	
5000	2743.7697(5)	36435.430	2743.76993(14)	–0.0002	4d^9^_3/2_5p	^2^[1/2]°_1_	4d^9^_5/2_5d	^2^[3/2]_1_	K02c	1.0e+08	D+	TW	
590	2728.73(4)	36636.3	2728.77443(13)	–0.05	4d^9^_3/2_5p	^2^[3/2]°_2_	4d^9^_5/2_5d	^2^[5/2]_2_	B30	1.0e+06	E	C05	
91000	2711.87305(22)	36863.955	2711.87292(13)	0.00013	4d^9^_5/2_5p	^2^[7/2]°_4_	4d^9^_5/2_6s	^2^[5/2]_3_	K02c	2.0e+08	C+	C05	
8900	2711.07823(22)	36874.762	2711.07830(12)	–0.00007	4d^9^_3/2_5p	^2^[3/2]°_1_	4d^9^_5/2_5d	^2^[3/2]_1_	K02c	4.8e+07	D+	TW	
2300	2688.1976(6)	37188.605	2688.19709(13)	0.0005	4d^9^_3/2_5p	^2^[1/2]°_1_	4d^9^_5/2_5d	^2^[5/2]_2_	K02c	1.40e+07	B+	C05	
24000	2681.19774(22)	37285.688	2681.19759(10)	0.00015	4d^9^_5/2_5p	^2^[3/2]°_1_	4d^9^_5/2_6s	^2^[5/2]_2_	K02c	6.0e+07	D+	TW	
2100	2669.1994(5)	37453.282	2669.1992(4)	0.0001	4d^8^5s^2^	^3^F_3_	4d^8^(^3^F)5s5p(^3^P°)	^5^G°_2_	K02c				
39000	2663.17552(21)	37537.993			4d^8^5s^2^	^3^F_4_	4d^8^(^3^F)5s5p(^3^P°)	^5^G°_5_	K02c				S
26000	2660.44956(21)	37576.453	2660.44959(9)	–0.00002	4d^9^_3/2_5s	^2^[3/2]_2_	4d^9^_5/2_5p	^2^[3/2]°_1_	K02c	1.51e+07	C+	F95c	
7000	2657.4137(3)	37619.379	2657.4137(3)	–0.0001	4d^8^5s^2^	^3^F_2_	4d^9^_3/2_6p	^2^[3/2]°_1_	K02c				
6200	2656.80859(21)	37627.946	2656.80863(11)	–0.00004	4d^9^_3/2_5p	^2^[3/2]°_1_	4d^9^_5/2_5d	^2^[5/2]_2_	K02c	2.90e+07	B+	C05	
2500	2656.5404(4)	37631.745	2656.54043(13)	–0.0001	4d^9^_3/2_5p	^2^[5/2]°_3_	4d^9^_5/2_5d	^2^[3/2]_2_	K02c	1.3e+07	D+	TW	
8200	2625.51589(21)	38076.395	2625.51592(10)	–0.00003	4d^9^_3/2_5p	^2^[5/2]°_3_	4d^9^_5/2_5d	^2^[5/2]_3_	K02c	3.00e+07	B+	C05	
20	2624.947(6)	38084.64	2624.9459(10)	0.002	4d^8^5s^2^	^3^F_4_	4d^9^_5/2_6p	^2^[7/2]°_3_	R40bc				
3600	2617.0020(3)	38200.261	2617.00202(21)	0.0000	4d^9^_3/2_5p	^2^[1/2]°_1_	4d^9^_5/2_5d	^2^[1/2]_0_	K02c	6.9e+07	B+	C05	
21000	2614.39776(21)	38238.311	2614.39778(10)	–0.00002	4d^9^_3/2_5p	^2^[5/2]°_2_	4d^9^_3/2_6s	^2^[3/2]_1_	K02c	1.36e+08	C+	C05	
24000	2605.97631(20)	38361.874	2605.97632(10)	–0.00001	4d^9^_5/2_5p	^2^[7/2]°_3_	4d^9^_5/2_6s	^2^[5/2]_3_	K02c	3.0e+07	C+	C05	
3400	2598.36(5)	38474.3	2598.38439(15)	–0.02	4d^9^_3/2_5p	^2^[5/2]°_3_	4d^9^_5/2_5d	^2^[7/2]_4_	S28c	8.0e+06	C+	C05	
24000	2596.84600(20)	38496.743	2596.84607(20)	–0.00007	4d^8^5s^2^	^3^F_4_	4d^9^_5/2_6p	^2^[7/2]°_4_	K02c				
8100	2595.49487(20)	38516.782	2595.49481(9)	0.00005	4d^9^_3/2_5p	^2^[5/2]°_2_	4d^9^_3/2_6s	^2^[3/2]_2_	K02c	2.8e+07	C+	C05	
1100	2587.24(4)	38639.7	2587.24477(20)	–0.01	4d^9^_3/2_5p	^2^[3/2]°_1_	4d^9^_5/2_5d	^2^[1/2]_0_	B30	2.0e+06	E	C05	
1400	2584.0917(10)	38686.739	2584.09063(20)	0.0011	4d^9^_3/2_5p	^2^[5/2]°_2_	4d^9^_5/2_5d	^2^[1/2]_1_	K02c				
80000	2580.57399(20)	38739.472	2580.57394(10)	0.00005	4d^9^_5/2_5p	^2^[7/2]°_3_	4d^9^_5/2_6s	^2^[5/2]_2_	K02c	1.8e+08	C	TW	
6400	2567.00519(20)	38944.230	2567.00514(9)	0.00005	4d^9^_5/2_5p	^2^[5/2]°_3_	4d^9^_3/2_6s	^2^[3/2]_2_	K02c	2.5e+07	C+	C05	
12000	2564.23332(20)	38986.325	2564.23336(12)	–0.00004	4d^9^_3/2_5p	^2^[1/2]°_0_	4d^9^_3/2_6s	^2^[3/2]_1_	K02c	6.2e+07	C+	C05	
66000	2557.48774(20)	39089.148	2557.48780(21)	–0.00007	4d^8^5s^2^	^3^F_4_	4d^9^_5/2_6p	^2^[5/2]°_3_	K02c				
2800	2553.4021(7)	39151.689	2553.40179(9)	0.0003	4d^9^_3/2_5s	^2^[3/2]_2_	4d^9^_5/2_5p	^2^[5/2]°_2_	K02c	7.e+05	E	F95c	
19000	2535.30785(19)	39431.093	2535.30785(8)	0.00000	4d^9^_5/2_5s	^2^[5/2]_2_	4d^9^_5/2_5p	^2^[3/2]°_2_	K02c	3.4e+06	E	F95c	
390	2535.07(4)	39434.8	2535.07148(19)	0.00	4d^9^_3/2_5p	^2^[1/2]°_0_	4d^9^_5/2_5d	^2^[1/2]_1_	B30				
6300	2532.6045(6)	39473.180	2532.6044(6)	0.0001	4d^8^5s^2^	^3^F _3_	4d^8^(^3^F)5s5p(^3^P°)	^5^F°_3_	K02c				
4300	2530.4869(5)	39506.210	2530.4865(4)	0.0004	4d^8^5s^2^	^3^F_3_	4d^9^_3/2_6p	^2^[3/2]°_2_	K02c				
12000	2507.0909(3)	39874.855	2507.09088(11)	0.0000	4d^9^_3/2_5p	^2^[3/2]°_2_	4d^9^_3/2_5d	^2^[1/2]_1_	K02c	9.e+07	D+	TW	
290	2506.91(4)	39877.8	2506.86992(12)	0.04	4d^9^_3/2_5p	^2^[5/2]°_2_	4d^9^_5/2_5d	^2^[3/2]_2_	B30				
5300	2506.6289(3)	39882.204	2506.62912(10)	–0.0003	4d^9^_3/2_5p	^2^[5/2]°_2_	4d^9^_5/2_5d	^2^[3/2]_1_	K02c	4.6e+07	D+	TW	
50000	2506.59265(19)	39882.780	2506.59265(9)	–0.00001	4d^9^_3/2_5s	^2^[3/2]_1_	4d^9^_5/2_5p	^2^[3/2]°_1_	K02c	2.13e+07	B	F95c	
17000	2503.90077(19)	39925.654	2503.90081(9)	–0.00004	4d^9^_5/2_5p	^2^[5/2]°_2_	4d^9^_3/2_6s	^2^[3/2]_1_	K02c	9.2e+07	B	C05	
5700	2486.55629(19)	40204.128	2486.55645(9)	–0.00015	4d^9^_5/2_5p	^2^[5/2]°_2_	4d^9^_3/2_6s	^2^[3/2]_2_	K02c	1.60e+07	C+	C05	
9300	2485.65135(25)	40218.764	2485.65135(12)	0.00000	4d^9^_5/2_5p	^2^[5/2]°_3_	4d^9^_5/2_5d	^2^[9/2]_4_	K02c	2.8e+07	D+	TW	
29000	2480.28243(18)	40305.817	2480.28243(11)	0.00000	4d^9^_5/2_5p	^2^[5/2]°_3_	4d^9^_5/2_5d	^2^[3/2]_2_	K02c	1.6e+08	C	TW	
1900	2479.2251(4)	40323.005	2479.22422(10)	0.0009	4d^9^_3/2_5p	^2^[5/2]°_2_	4d^9^_5/2_5d	^2^[5/2]_3_	K02c	3.0e+06	E	C05	
100000	2477.12768(18)	40357.145	2477.12763(9)	0.00004	4d^9^_5/2_5p	^2^[3/2]°_2_	4d^9^_5/2_6s	^2^[5/2]_3_	K02c	1.4e+08	C	C05	
7200	2476.61(5)	40365.5	2476.66649(13)	–0.05	4d^9^_3/2_5p	^2^[3/2]°_2_	4d^9^_3/2_5d	^2^[7/2]_3_	S28c	2.2e+07	D+	TW	
3600	2476.09(5)	40374.1	2476.08744(18)	0.00	4d^9^_5/2_5p	^2^[5/2]°_2_	4d^9^_5/2_5d	^2^[1/2]_1_	S28c				
140000	2473.79016(18)	40411.589	2473.79016(9)	0.00001	4d^9^_3/2_5s	^2^[3/2]_2_	4d^9^_5/2_5p	^2^[5/2]°_3_	K02c	4.98e+07	A	F95c	
9700	2472.79642(24)	40427.828	2472.79659(12)	–0.00017	4d^9^_3/2_5p	^2^[1/2]°_1_	4d^9^_3/2_5d	^2^[1/2]_1_	K02c	2.3e+08	D+	TW	
35000	2462.12773(18)	40602.994	2462.12763(10)	0.00010	4d^9^_3/2_5p	^2^[5/2]°_2_	4d^9^_5/2_5d	^2^[7/2]_3_	K02c	8.e+07	D+	TW	
5500	2461.1412(3)	40619.269	2461.14111(11)	0.0000	4d^9^_3/2_5p	^2^[3/2]°_2_	4d^9^_3/2_5d	^2^[3/2]_1_	K02c	3.4e+07	D+	TW	
34000	2460.16512(18)	40635.383	2460.16505(9)	0.00007	4d^9^_3/2_5p	^2^[5/2]°_2_	4d^9^_5/2_5d	^2^[5/2]_2_	K02c	1.18e+08	B+	C05	
680	2454.1636(4)	40734.747	2454.16376(9)	–0.0001	4d^9^_5/2_5p	^2^[3/2]°_2_	4d^9^_5/2_6s	^2^[5/2]_2_	K02c				
110000	2453.21676(18)	40750.468	2453.21679(8)	–0.00003	4d^9^_5/2_5p	^2^[5/2]°_3_	4d^9^_5/2_5d	^2^[5/2]_3_	K02c	3.7e+08	B+	C05	
210000	2447.89606(18)	40839.036	2447.89600(8)	0.00006	4d^9^_3/2_5s	^2^[3/2]_2_	4d^9^_3/2_5p	^2^[5/2]°_2_	K02c	9.2e+07	B+	F95c	
22000	2446.21146(18)	40867.158	2446.21139(11)	0.00007	4d^9^_3/2_5p	^2^[3/2]°_1_	4d^9^_3/2_5d	^2^[1/2]_1_	K02c	3.5e+07	D+	TW	
140000	2444.09896(18)	40902.478	2444.09897(10)	–0.00001	4d^9^_3/2_5p	^2^[3/2]°_2_	4d^9^_3/2_5d	^2^[3/2]_2_	K02c	2.7e+08	C	TW	
450000	2437.78299(18)	41008.443	2437.78303(9)	–0.00004	4d^9^_5/2_5s	^2^[5/2]_3_	4d^9^_5/2_5p	^2^[3/2]°_2_	K02c	2.90e+08	B	F95c	
12000	2436.47567(24)	41030.445	2436.47576(10)	–0.00009	4d^9^_5/2_5p	^2^[5/2]°_3_	4d^9^_5/2_5d	^2^[7/2]_3_	K02c				
190000	2429.51289(18)	41148.026	2429.51294(12)	–0.00005	4d^9^_5/2_5p	^2^[5/2]°_3_	4d^9^_5/2_5d	^2^[7/2]_4_	K02c	4.0e+08	B+	C05	
30000	2428.08389(18)	41172.241	2428.08393(11)	–0.00004	4d^9^_3/2_5p	^2^[1/2]°_1_	4d^9^_3/2_5d	^2^[3/2]_1_	K02c				
41000	2422.46274(18)	41267.771	2422.46271(9)	0.00003	4d^9^_3/2_5p	^2^[3/2]°_2_	4d^9^_3/2_5d	^2^[5/2]_2_	K02c	5.2e+07	D+	TW	
540000	2419.95667(18)	41310.504	2419.95662(11)	0.00005	4d^9^_3/2_5p	^2^[3/2]°_2_	4d^9^_3/2_5d	^2^[5/2]_3_	K02c	4.6e+08	C	TW	
470000	2413.18810(17)	41426.364	2413.18805(8)	0.00005	4d^9^_5/2_5s	^2^[5/2]_2_	4d^9^_5/2_5p	^2^[7/2]°_3_	K02c	2.10e+08	B+	F95c	
91000	2411.49494(17)	41455.448	2411.49491(10)	0.00003	4d^9^_3/2_5p	^2^[1/2]°_1_	4d^9^_3/2_5d	^2^[3/2]_2_	K02c	2.5e+08	C	TW	
180000	2411.34527(17)	41458.021	2411.34524(8)	0.00003	4d^9^_3/2_5s	^2^[3/2]_1_	4d^9^_5/2_5p	^2^[5/2]°_2_	K02c	9.6e+07	B+	F95c	
5900	2408.85401(17)	41500.894	2408.85395(8)	0.00006	4d^9^_5/2_5p	^2^[3/2]°_1_	4d^9^_3/2_6s	^2^[3/2]_1_	K02c	1.7e+07	C+	C05	
15000	2404.87583(17)	41569.540	2404.87569(9)	0.00014	4d^9^_5/2_5p	^2^[5/2]°_2_	4d^9^_5/2_5d	^2^[3/2]_1_	K02c	1.1e+08	D+	TW	
31000	2402.44630(17)	41611.575	2402.44634(10)	–0.00004	4d^9^_3/2_5p	^2^[3/2]°_1_	4d^9^_3/2_5d	^2^[3/2]_1_	K02c	3.6e+08	C	TW	
11000	2392.79686(17)	41779.369	2392.79697(8)	–0.00011	4d^9^_5/2_5p	^2^[3/2]°_1_	4d^9^_3/2_6s	^2^[3/2]_2_	K02c	2.50e+07	B	C05	
310000	2390.42949(17)	41820.742	2390.42948(9)	0.00001	4d^9^_3/2_5p	^2^[1/2]°_1_	4d^9^_3/2_5d	^2^[5/2]_2_	K02c	6.8e+07	D+	TW	
32000	2386.20459(17)	41894.782	2386.20456(9)	0.00003	4d^9^_3/2_5p	^2^[3/2]°_1_	4d^9^_3/2_5d	^2^[3/2]_2_	K02c				
14000	2383.10087(23)	41949.341	2383.10093(14)	–0.00006	4d^9^_5/2_5p	^2^[3/2]°_1_	4d^9^_5/2_5d	^2^[1/2]_1_	K02c	8.e+07	D+	TW	
2800	2379.60(5)	42011.0	2379.63907(9)	–0.04	4d^9^_5/2_5p	^2^[5/2]°_2_	4d^9^_5/2_5d	^2^[5/2]_3_	S28c	9.e+05	E	C05	
16000	2373.5695(3)	42117.781	2373.56965(12)	–0.0002	4d^9^_3/2_5p	^2^[5/2]°_3_	4d^9^_3/2_5d	^2^[7/2]_3_	K02c	3.7e+07	D+	TW	
9800	2370.3984(4)	42174.121	2370.3981(4)	0.0003	4d^8^5s^2^	^3^F_4_	4d^8^(^3^F)5s5p(^3^P°)	^5^F°_4_	K02c				
100000	2365.57671(17)	42260.076	2365.57671(8)	0.00001	4d^9^_3/2_5p	^2^[3/2]°_1_	4d^9^_3/2_5d	^2^[5/2]_2_	K02c	3.0e+08	C	TW	
100000	2363.88377(17)	42290.339	2363.88381(10)	–0.00004	4d^9^_5/2_5p	^2^[5/2]°_2_	4d^9^_5/2_5d	^2^[7/2]_3_	K02c	3.7e+08	C	TW	
67000	2362.07463(17)	42322.727	2362.07464(9)	–0.00001	4d^9^_5/2_5p	^2^[5/2]°_2_	4d^9^_5/2_5d	^2^[5/2]_2_	K02c	2.63e+08	B+	C05	
260000	2358.75114(17)	42382.355	2358.75114(17)	0.00000	4d^9^_3/2_5p	^2^[5/2]°_3_	4d^9^_3/2_5d	^2^[7/2]_4_	K02c	6.7e+08	C	TW	
170000	2357.91689(17)	42397.349	2357.91680(9)	0.00008	4d^9^_3/2_5s	^2^[3/2]_1_	4d^9^_3/2_5p	^2^[1/2]°_0_	K02c	2.96e+08	A	B97	
14000	2343.64027(22)	42655.597	2343.64032(9)	–0.00004	4d^9^_3/2_5p	^2^[5/2]°_3_	4d^9^_3/2_5d	^2^[3/2]_2_	K02c				
330000	2331.36632(16)	42880.147	2331.36629(7)	0.00003	4d^9^_5/2_5s	^2^[5/2]_2_	4d^9^_5/2_5p	^2^[3/2]°_1_	K02c	2.51e+08	B	F95c	
270000	2324.94655(16)	42998.540	2324.94655(16)	–0.00001	4d^9^_5/2_5p	^2^[7/2]°_4_	4d^9^_5/2_5d	^2^[9/2]_5_	K02c	7.1e+08	C	TW	
240000	2324.66685(16)	43003.713	2324.66678(8)	0.00007	4d^9^_5/2_5s	^2^[5/2]_3_	4d^9^_5/2_5p	^2^[7/2]°_3_	K02c	8.4e+07	B+	F95c	
5400	2324.33945(22)	43009.770	2324.33966(12)	–0.00022	4d^9^_5/2_5p	^2^[7/2]°_4_	4d^9^_5/2_5d	^2^[9/2]_4_	K02c	3.5e+07	D+	TW	
34000	2323.73860(16)	43020.890	2323.73861(8)	–0.00001	4d^9^_3/2_5p	^2^[5/2]°_3_	4d^9^_3/2_5d	^2^[5/2]_2_	K02c	2.0e+07	D+	TW	
190000	2321.43261(16)	43063.621	2321.43249(10)	0.00012	4d^9^_3/2_5p	^2^[5/2]°_3_	4d^9^_3/2_5d	^2^[5/2]_3_	K02c	1.8e+08	C	TW	
730000	2320.24495(16)	43085.662	2320.24495(8)	0.00000	4d^9^_3/2_5s	^2^[3/2]_2_	4d^9^_3/2_5p	^2^[5/2]°_3_	K02c	2.90e+08	B	F95c	
490	2317.26(3)	43141.1	2317.27097(10)	–0.01	4d^9^_5/2_5p	^2^[3/2]°_1_	4d^9^_5/2_5d	^2^[3/2]_2_	B30				
27000	2317.06532(16)	43144.782	2317.06521(9)	0.00011	4d^9^_5/2_5p	^2^[3/2]°_1_	4d^9^_5/2_5d	^2^[3/2]_1_	K02c	2.5e+08	D+	TW	
340000	2317.03406(16)	43145.364	2317.03405(8)	0.00001	4d^9^_3/2_5s	^2^[3/2]_1_	4d^9^_3/2_5p	^2^[5/2]°_2_	K02c	1.68e+08	B+	F95c	
3800	2312.32(5)	43233.3	2312.32886(8)	–0.01	4d^9^_5/2_5p	^2^[7/2]°_3_	4d^9^_3/2_6s	^2^[3/2]_2_	S28c	1.0e+06	D	C05	
27000	2295.95366(16)	43541.470	2295.95369(9)	–0.00003	4d^9^_5/2_5p	^2^[7/2]°_4_	4d^9^_5/2_5d	^2^[5/2]_3_	K02c	6.0e+07	B+	C05	
590000	2279.98111(16)	43846.475	2279.98107(7)	0.00004	4d^9^_3/2_5s	^2^[3/2]_2_	4d^9^_3/2_5p	^2^[3/2]°_1_	K02c	3.25e+08	B+	F95c1	
41000	2277.30636(16)	43897.969	2277.30639(8)	–0.00003	4d^9^_5/2_5p	^2^[3/2]°_1_	4d^9^_5/2_5d	^2^[5/2]_2_	K02c	1.50e+08	B+	C05	Las
120000	2275.17818(16)	43939.027	2275.17817(10)	0.00001	4d^9^_5/2_5p	^2^[7/2]°_4_	4d^9^_5/2_5d	^2^[7/2]_4_	K02c	2.16e+08	B+	C05	
11000	2265.7893(4)	44121.082	2265.78950(12)	–0.0002	4d^9^_3/2_5p	^2^[1/2]°_1_	4d^9^_3/2_5d	^2^[1/2]_0_	K02c	1.9e+08	D+	TW	
4600	2257.34(4)	44286.2	2257.36061(8)	–0.02	4d^9^_3/2_5s	^2^[3/2]_2_	4d^9^_3/2_5p	^2^[1/2]°_1_	S28c	2.2e+08	D+	B97	
130000	2253.36127(15)	44364.402	2253.36124(10)	0.00004	4d^9^_3/2_5p	^2^[5/2]°_2_	4d^9^_3/2_5d	^2^[7/2]_3_	K02c	5.6e+08	C	TW	
460000	2248.74892(15)	44455.388	2248.74886(7)	0.00006	4d^9^_5/2_5s	^2^[5/2]_2_	4d^9^_5/2_5p	^2^[5/2]°_2_	K02c	2.57e+08	B+	F95c	
900000	2246.41186(15)	44501.633	2246.41194(9)	–0.00008	4d^9^_5/2_5s	^2^[5/2]_3_	4d^9^_5/2_5p	^2^[7/2]°_4_	K02c	3.91e+08	A	B97	
110000	2246.10652(15)	44507.682	2246.10646(10)	0.00006	4d^9^_5/2_5p	^2^[7/2]°_3_	4d^9^_5/2_5d	^2^[9/2]_4_	K02c	7.1e+08	C	TW	
2400000	2243.44841(15)	44560.411	2243.44833(11)	0.00009	4d^9^_3/2_5p	^2^[3/2]°_1_	4d^9^_3/2_5d	^2^[1/2]_0_	K02c	4.3e+08	D+	TW	Las
10000	2241.72134(20)	44594.738	2241.72144(9)	–0.00010	4d^9^_5/2_5p	^2^[7/2]°_3_	4d^9^_5/2_5d	^2^[3/2]_2_	K02c	4.7e+07	D+	TW	
6900	2240.5016(3)	44619.013	2240.50161(9)	0.0000	4d^9^_3/2_5p	^2^[5/2]°_2_	4d^9^_3/2_5d	^2^[3/2]_1_	K02c	4.2e+07	D+	TW	
34000	2240.32094(15)	44622.611	2240.32091(10)	0.00003	4d^9^_3/2_5p	^2^[1/2]°_0_	4d^9^_3/2_5d	^2^[1/2]_1_	K02c	1.9e+08	D+	TW	
520000	2229.51924(15)	44838.780	2229.51923(7)	0.00001	4d^9^_3/2_5s	^2^[3/2]_2_	4d^9^_3/2_5p	^2^[3/2]°_2_	K02c	2.09e+08	B+	F95c	
35000	2226.00217(15)	44909.618	2226.00217(12)	0.00000	4d^9^_5/2_5p	^2^[3/2]°_1_	4d^9^_5/2_5d	^2^[1/2]_0_	K02c	6.1e+08	B	C05	
			2224.00468(8)		4d^9^_5/2_5p	^2^[3/2]°_2_	4d^9^_3/2_6s	^2^[3/2]_1_		4.0e+06	D	C05	P
34000	2219.58790(15)	45039.387	2219.58792(7)	–0.00002	4d^9^_5/2_5p	^2^[7/2]°_3_	4d^9^_5/2_5d	^2^[5/2]_3_	K02c	7.3e+07	B+	C05	
9100	2218.7484(3)	45056.426	2218.74835(15)	0.0001	4d^9^_5/2_5p	^2^[5/2]°_3_	4d^9^_3/2_5d	^2^[7/2]_4_	K02c				
2000	2210.31012(24)	45228.421	2210.31008(7)	0.00003	4d^9^_5/2_5p	^2^[3/2]°_2_	4d^9^_3/2_6s	^2^[3/2]_2_	K02c	3.00e+06	B+	C05	
160000	2208.40105(15)	45267.515	2208.40106(7)	–0.00001	4d^9^_3/2_5p	^2^[5/2]°_2_	4d^9^_3/2_5d	^2^[5/2]_2_	K02c	1.9e+08	D+	TW	
120000	2205.87431(15)	45319.362	2205.87428(8)	0.00003	4d^9^_5/2_5p	^2^[7/2]°_3_	4d^9^_5/2_5d	^2^[7/2]_3_	K02c	2.5e+08	C	TW	
26000	2204.29872(19)	45351.752	2204.29876(8)	–0.00004	4d^9^_5/2_5p	^2^[7/2]°_3_	4d^9^_5/2_5d	^2^[5/2]_2_	K02c	5.6e+07	C+	C05	
63000	2203.55651(15)	45367.026	2203.55647(9)	0.00003	4d^9^_3/2_5p	^2^[1/2]°_0_	4d^9^_3/2_5d	^2^[3/2]_1_	K02c	2.1e+08	D+	TW	
120000	2202.03376(15)	45398.395	2202.03378(11)	–0.00002	4d^9^_5/2_5p	^2^[3/2]°_2_	4d^9^_5/2_5d	^2^[1/2]_1_	K02c	6.7e+08	C	TW	
			2200.16534(11)		4d^9^_5/2_5p	^2^[7/2]°_3_	4d^9^_5/2_5d	^2^[7/2]_4_		3.0e+06	E	C05	P
210000	2186.76813(14)	45715.284	2186.76813(6)	0.00000	4d^9^_5/2_5s	^2^[5/2]_2_	4d^9^_5/2_5p	^2^[5/2]°_3_	K02c	7.27e+07	A	F95c	
10000	2185.69653(19)	45737.695	2185.69647(10)	0.00005	4d^9^_5/2_5p	^2^[5/2]°_3_	4d^9^_3/2_5d	^2^[5/2]_3_	K02c				
28000	2171.6858(4)	46032.743	2171.68597(8)	–0.0002	4d^9^_5/2_5s	^2^[5/2]_3_	4d^9^_5/2_5p	^2^[5/2]°_2_	K02c	3.9e+06	E	B97	
84000	2170.78958(14)	46051.745	2170.78956(10)	0.00002	4d^9^_5/2_5p	^2^[5/2]°_2_	4d^9^_3/2_5d	^2^[7/2]_3_	K02c	1.6e+08	C	TW	
150000	2166.50867(14)	46142.731	2166.50863(6)	0.00004	4d^9^_5/2_5s	^2^[5/2]_2_	4d^9^_3/2_5p	^2^[5/2]°_2_	K02c	6.0e+07	B	F95c	
22000	2166.0359(3)	46152.802	2166.03582(7)	0.0000	4d^9^_3/2_5s	^2^[3/2]_1_	4d^9^_3/2_5p	^2^[3/2]°_1_	K02c	1.7e+08	D+	TW	
4700	2158.8525(4)	46306.353	2158.85242(9)	0.0001	4d^9^_5/2_5p	^2^[5/2]°_2_	4d^9^_3/2_5d	^2^[3/2]_1_	K02c				
75000	2145.70735(14)	46590.007	2145.70730(8)	0.00005	4d^9^_5/2_5p	^2^[3/2]°_2_	4d^9^_5/2_5d	^2^[3/2]_2_	K02c	5.2e+08	C	TW	
390000	2145.60920(14)	46592.138	2145.60925(7)	–0.00006	4d^9^_3/2_5s	^2^[3/2]_1_	4d^9^_3/2_5p	^2^[1/2]°_1_	K02c	3.78e+08	B+	F95c1	
6000	2145.53095(18)	46593.837	2145.53088(8)	0.00007	4d^9^_5/2_5p	^2^[3/2]°_2_	4d^9^_5/2_5d	^2^[3/2]_1_	K02c	1.3e+08	D+	TW	
64000	2129.03277(14)	46954.858	2129.03279(7)	–0.00002	4d^9^_5/2_5p	^2^[5/2]°_2_	4d^9^_3/2_5d	^2^[5/2]_2_	K02c	7.6e+07	D+	TW	
66000	2125.42019(14)	47034.658	2125.42016(7)	0.00002	4d^9^_5/2_5p	^2^[3/2]°_2_	4d^9^_5/2_5d	^2^[5/2]_3_	K02c	1.47e+08	B+	C05	
6900	2120.79683(18)	47137.182	2120.79682(8)	0.00001	4d^9^_5/2_5p	^2^[3/2]°_1_	4d^9^_3/2_5d	^2^[1/2]_1_	K02c	1.5e+08	D+	TW	
220000	2120.44020(13)	47145.109	2120.44026(6)	–0.00007	4d^9^_3/2_5s	^2^[3/2]_1_	4d^9^_3/2_5p	^2^[3/2]°_2_	K02c	1.19e+08	B+	F95c	
530000	2113.82490(13)	47292.634	2113.82492(7)	–0.00003	4d^9^_5/2_5s	^2^[5/2]_3_	4d^9^_5/2_5p	^2^[5/2]°_3_	K02c	3.01e+08	A	F95c	
4100	2111.40(3)	47347.0	2111.39639(8)	0.00	4d^9^_5/2_5p	^2^[3/2]°_2_	4d^9^_5/2_5d	^2^[5/2]_2_	S28c	1.00e+07	B	C05	
140	2100.75(9)	47587.0	2100.93065(13)	–0.18	4d^9^_5/2_5p	^2^[7/2]°_4_	4d^9^_3/2_5d	^2^[7/2]_3_	G35c				
800			2094.88826(7)		4d^9^_5/2_5s	^2^[5/2]_3_	4d^9^_3/2_5p	^2^[5/2]°_2_	F95	4.8e+05	D+	F95c	P
740	2089.318(22)	47847.3	2089.31191(13)	0.006	4d^9^_5/2_5p	^2^[7/2]°_4_	4d^9^_3/2_5d	^2^[7/2]_4_	G35c				
2300	2087.8208(4)	47881.598	2087.82083(8)	0.0000	4d^9^_5/2_5p	^2^[3/2]°_1_	4d^9^_3/2_5d	^2^[3/2]_1_	K02c				
36000	2075.54292(13)	48164.806	2075.54294(7)	–0.00002	4d^9^_5/2_5p	^2^[3/2]°_1_	4d^9^_3/2_5d	^2^[3/2]_2_	K02c	1.2e+08	D+	TW	
120000	2065.91011(13)	48389.357	2065.91012(6)	0.00000	4d^9^_5/2_5s	^2^[5/2]_2_	4d^9^_3/2_5p	^2^[5/2]°_3_	K02c	4.7e+07	B+	F95c	
540	2059.939(21)	48529.6	2059.97746(10)	–0.038	4d^9^_5/2_5p	^2^[7/2]°_4_	4d^9^_3/2_5d	^2^[5/2]_3_	G35c				
38000	2033.92680(12)	49150.172	2033.92686(5)	–0.00006	4d^9^_5/2_5s	^2^[5/2]_2_	4d^9^_3/2_5p	^2^[3/2]°_1_	K02c	2.4e+07	B	F95c1	
570	2025.858(21)	49345.9	2025.88091(12)	–0.023	4d^9^_5/2_5p	^2^[7/2]°_3_	4d^9^_3/2_5d	^2^[7/2]_4_	G35c				
34000	2015.90481(12)	49589.507	2015.90491(6)	–0.00010	4d^9^_5/2_5s	^2^[5/2]_2_	4d^9^_3/2_5p	^2^[1/2]°_1_	K02c	3.7e+07	C+	F95c1	
40000	2000.68462(12)	49966.703	2000.68449(6)	0.00013	4d^9^_5/2_5s	^2^[5/2]_3_	4d^9^_3/2_5p	^2^[5/2]°_3_	K02c	1.75e+07	B+	F95c	
730	1998.9350(8)	50026.640	1998.93596(8)	–0.0010	4d^9^_5/2_5p	^2^[7/2]°_3_	4d^9^_3/2_5d	^2^[5/2]_3_	K02c				
140000	1994.31719(12)	50142.475	1994.31719(5)	0.00001	4d^9^_5/2_5s	^2^[5/2]_2_	4d^9^_3/2_5p	^2^[3/2]°_2_	K02c	4.08e+07	B+	F95c	
24000	1967.32521(12)	50830.437	1967.32526(8)	–0.00005	4d^9^_5/2_5p	^2^[3/2]°_1_	4d^9^_3/2_5d	^2^[1/2]_0_	K02c				
260	1937.455(19)	51614.1	1937.46408(7)	–0.009	4d^9^_5/2_5p	^2^[3/2]°_2_	4d^9^_3/2_5d	^2^[3/2]_2_	G35c				
22000	1933.49455(11)	51719.825	1933.49457(6)	–0.00001	4d^9^_5/2_5s	^2^[5/2]_3_	4d^9^_3/2_5p	^2^[3/2]°_2_	K02c	2.3e+07	C+	F95c	
2200	1922.2672(3)	52021.905	1922.26783(8)	–0.0007	4d^9^_5/2_5p	^2^[3/2]°_2_	4d^9^_3/2_5d	^2^[5/2]_3_	K02c				
180	1865.98(3)	53591.2	1865.999(3)	–0.02	4d^9^_3/2_5p	^2^[3/2]°_2_	4d^9^_5/2_7s	^2^[5/2]_3_	G35c				
93	1860.88(10)	53738	1860.9388(24)	–0.06	4d^9^_3/2_5p	^2^[3/2]°_2_	4d^9^_5/2_7s	^2^[5/2]_2_	G35c				
210	1841.99(3)	54289.0	1841.9840(24)	0.01	4d^9^_3/2_5p	^2^[1/2]°_1_	4d^9^_5/2_7s	^2^[5/2]_2_	G35c				
1100	1827.18(3)	54729.1	1827.1975(23)	–0.02	4d^9^_3/2_5p	^2^[3/2]°_1_	4d^9^_5/2_7s	^2^[5/2]_2_	G35c				
780	1806.87(3)	55344.3	1806.890(3)	–0.02	4d^9^_3/2_5p	^2^[5/2]°_3_	4d^9^_5/2_7s	^2^[5/2]_3_	G35c				
1400	1802.19(3)	55488.0	1802.1448(23)	0.05	4d^9^_3/2_5p	^2^[5/2]°_3_	4d^9^_5/2_7s	^2^[5/2]_2_	G35c				
1900	1732.017(3)	57736.16	1732.0198(21)	–0.003	4d^9^_3/2_5p	^2^[5/2]°_2_	4d^9^_5/2_7s	^2^[5/2]_2_	K02c				
5800	1723.612(3)	58017.70	1723.609(3)	0.003	4d^9^_5/2_5p	^2^[5/2]°_3_	4d^9^_5/2_7s	^2^[5/2]_3_	K02c	3.6e+07	D+	TW	
2200	1719.295(3)	58163.37	1719.2910(21)	0.004	4d^9^_5/2_5p	^2^[5/2]°_3_	4d^9^_5/2_7s	^2^[5/2]_2_	K02c				
920	1718.360(3)	58195.01	1718.360(3)	0.000	4d^9^_3/2_5p	^2^[3/2]°_2_	4d^9^_3/2_7s	^2^[3/2]_1_	K02c				
1500	1715.44(3)	58294.1	1715.4325(24)	0.01	4d^9^_3/2_5p	^2^[3/2]°_2_	4d^9^_3/2_7s	^2^[3/2]_2_	G35c	3.4e+07	D+	TW	
4000	1702.185(3)	58748.02	1702.186(3)	–0.001	4d^9^_3/2_5p	^2^[1/2]°_1_	4d^9^_3/2_7s	^2^[3/2]_1_	K02c				
2200	1686.718(3)	59286.75	1686.7206(23)	–0.003	4d^9^_3/2_5p	^2^[3/2]°_1_	4d^9^_3/2_7s	^2^[3/2]_2_	K02c				
8000	1682.835(3)	59423.52	1682.8386(20)	–0.003	4d^9^_5/2_5p	^2^[5/2]°_2_	4d^9^_5/2_7s	^2^[5/2]_2_	K02c	3.8e+07	D+	TW	
14000	1665.351(3)	60047.39	1665.3495(22)	0.002	4d^9^_3/2_5p	^2^[5/2]°_3_	4d^9^_3/2_7s	^2^[3/2]_2_	K02c	7.1e+07	D+	TW	
7700	1644.496(3)	60808.91	1644.4991(24)	–0.003	4d^9^_5/2_5p	^2^[7/2]°_4_	4d^9^_5/2_7s	^2^[5/2]_3_	K02c	7.1e+07	D+	TW	
2400	1639.382(3)	60998.59	1639.3807(19)	0.001	4d^9^_5/2_5p	^2^[3/2]°_1_	4d^9^_5/2_7s	^2^[5/2]_2_	K02c				
3700	1607.88(3)	62193.7	1607.8525(23)	0.03	4d^9^_3/2_5p	^2^[5/2]°_2_	4d^9^_3/2_7s	^2^[3/2]_1_	G35c	5.2e+07	D+	TW	
5000	1605.24(3)	62296.0	1605.2890(21)	–0.05	4d^9^_3/2_5p	^2^[5/2]°_2_	4d^9^_3/2_7s	^2^[3/2]_2_	G35c				
3500	1604.96(3)	62306.8	1604.9636(23)	0.00	4d^9^_5/2_5p	^2^[7/2]°_3_	4d^9^_5/2_7s	^2^[5/2]_3_	G35c				
7200	1601.24(3)	62451.6	1601.2188(18)	0.02	4d^9^_5/2_5p	^2^[7/2]°_3_	4d^9^_5/2_7s	^2^[5/2]_2_	G35c	6.3e+07	D+	TW	
6300	1594.27(3)	62724.5	1594.3489(20)	–0.08	4d^9^_5/2_5p	^2^[5/2]°_3_	4d^9^_3/2_7s	^2^[3/2]_2_	G35c				
3700	1588.77(3)	62941.6	1588.7447(23)	0.03	4d^9^_3/2_5p	^2^[1/2]°_0_	4d^9^_3/2_7s	^2^[3/2]_1_	G35c				
1200	1565.376(25)	63882.4	1565.3836(22)	–0.007	4d^9^_5/2_5p	^2^[5/2]°_2_	4d^9^_3/2_7s	^2^[3/2]_1_	G35c				
8400	1555.188(24)	64300.9	1555.1621(22)	0.026	4d^9^_5/2_5p	^2^[3/2]°_2_	4d^9^_5/2_7s	^2^[5/2]_3_	G35c	4.3e+07	D+	TW	
420	1551.69(7)	64446	1551.6458(17)	0.04	4d^9^_5/2_5p	^2^[3/2]°_2_	4d^9^_5/2_7s	^2^[5/2]_2_	G35c				
860	1532.21(7)	65265	1532.194(5)	0.02	4d^9^_3/2_5p	^2^[3/2]°_1_	4d^9^_5/2_8s	^2^[5/2]_2_	G35c				
540	1527.74(7)	65456	1527.7124(21)	0.03	4d^9^_5/2_5p	^2^[3/2]°_1_	4d^9^_3/2_7s	^2^[3/2]_1_	G35c				
1100	1525.434(23)	65555.1	1525.3979(19)	0.036	4d^9^_5/2_5p	^2^[3/2]°_1_	4d^9^_3/2_7s	^2^[3/2]_2_	G35c				
800	1514.55(7)	66026	1514.539(5)	0.02	4d^9^_3/2_5p	^2^[5/2]°_3_	4d^9^_5/2_8s	^2^[5/2]_2_	G35c				
5700	1492.268(22)	67012.1	1492.3046(18)	–0.037	4d^9^_5/2_5p	^2^[7/2]°_3_	4d^9^_3/2_7s	^2^[3/2]_2_	G35c				
1300	1457.121(21)	68628.5	1457.125(5)	–0.004	4d^9^_5/2_5p	^2^[5/2]°_3_	4d^9^_5/2_8s	^2^[5/2]_3_	G35c				
660	1455.67(6)	68697	1455.588(5)	0.08	4d^9^_5/2_5p	^2^[5/2]°_3_	4d^9^_5/2_8s	^2^[5/2]_2_	G35c				
900?	1453.23(6)	68812	1453.333(5)	–0.10	4d^9^_3/2_5p	^2^[3/2]°_2_	4d^9^_3/2_8s	^2^[3/2]_1_	G35c				
420	1451.32(6)	68903	1451.2440(19)	0.07	4d^9^_5/2_5p	^2^[3/2]°_2_	4d^9^_3/2_7s	^2^[3/2]_1_	G35c				
450	1449.1577(23)	69005.60	1449.1552(17)	0.0025	4d^9^_5/2_5p	^2^[3/2]°_2_	4d^9^_3/2_7s	^2^[3/2]_2_	K02c				
900	1441.77(6)	69359	1441.746(5)	0.03	4d^9^_3/2_5p	^2^[1/2]°_1_	4d^9^_3/2_8s	^2^[3/2]_1_	G35c				
910	1431.72(6)	69846	1431.745(5)	–0.02	4d^9^_3/2_5p	^2^[3/2]°_1_	4d^9^_3/2_8s	^2^[3/2]_2_	G35c				
650	1429.45(6)	69957	1429.375(4)	0.07	4d^9^_5/2_5p	^2^[5/2]°_2_	4d^9^_5/2_8s	^2^[5/2]_2_	G35c				
1900	1416.304(20)	70606.3	1416.318(5)	–0.013	4d^9^_3/2_5p	^2^[5/2]°_3_	4d^9^_3/2_8s	^2^[3/2]_2_	G35c				
2900	1400.194(20)	71418.7	1400.182(5)	0.012	4d^9^_5/2_5p	^2^[7/2]°_4_	4d^9^_5/2_8s	^2^[5/2]_3_	G35c	3.6e+07	D+	TW	
750	1397.96(6)	71533	1397.899(4)	0.06	4d^9^_5/2_5p	^2^[3/2]°_1_	4d^9^_5/2_8s	^2^[5/2]_2_	G35c				
1400	1373.55(6)	72804	1373.492(5)	0.06	4d^9^_3/2_5p	^2^[5/2]°_2_	4d^9^_3/2_8s	^2^[3/2]_1_	G35c				
1400	1372.61(6)	72854	1372.641(5)	–0.03	4d^9^_3/2_5p	^2^[5/2]°_2_	4d^9^_3/2_8s	^2^[3/2]_2_	G35c				
1000	1371.48(6)	72914	1371.418(4)	0.06	4d^9^_5/2_5p	^2^[7/2]°_3_	4d^9^_5/2_8s	^2^[5/2]_3_	G35c				
4200	1370.062(19)	72989.4	1370.057(4)	0.005	4d^9^_5/2_5p	^2^[7/2]°_3_	4d^9^_5/2_8s	^2^[5/2]_2_	G35c				
1600	1364.65(6)	73279	1364.634(4)	0.01	4d^9^_5/2_5p	^2^[5/2]°_3_	4d^9^_3/2_8s	^2^[3/2]_2_	G35c				
2400	1334.92(5)	74911	1334.891(4)	0.03	4d^9^_5/2_5p	^2^[3/2]°_2_	4d^9^_5/2_8s	^2^[5/2]_3_	G35c				
2200000	1195.8091(11)	83625.39	1195.8073(9)	0.0018	4d^10^	^1^S_0_	4d^9^_5/2_5p	^2^[3/2]°_1_	K02c	5.7e+07	D+	B97	
39	1180.9712(22)	84676.07	1180.9722(3)	–0.0010	4d^9^_3/2_5s	^2^[3/2]_2_	4d^8^(^3^F)5s5p(^3^P°)	^5^D°_3_	K02c				
160	1169.2079(10)	85527.99	1169.2083(10)	–0.0004	4d^9^_3/2_5s	^2^[3/2]_2_	4d^9^_5/2_6p	^2^[3/2]°_2_	K02c				
420	1163.2169(9)	85968.49	1163.21641(20)	0.0005	4d^9^_3/2_5s	^2^[3/2]_2_	4d^9^_5/2_6p	^2^[7/2]°_3_	K02c				
220	1152.164(13)	86793.2	1152.17144(7)	–0.007	4d^9^_3/2_5s	^2^[3/2]_2_	4d^9^_5/2_6p	^2^[3/2]°_1_	G35c				
290	1151.1284(9)	86871.28	1151.12847(7)	0.0000	4d^9^_3/2_5s	^2^[3/2]_2_	4d^9^_5/2_6p	^2^[5/2]°_2_	K02c				
250	1149.7824(9)	86972.98	1149.78197(4)	0.0004	4d^9^_3/2_5s	^2^[3/2]_2_	4d^9^_5/2_6p	^2^[5/2]°_3_	K02c				
130	1148.2009(9)	87092.77	1148.2007(4)	0.0002	4d^9^_3/2_5s	^2^[3/2]_2_	4d^8^(^3^F)5s5p(^3^P°)	^5^D°_2_	K02c				
65	1132.568(13)	88294.9	1132.57885(9)	–0.011	4d^9^_3/2_5s	^2^[3/2]_2_	4d^8^(^3^F)5s5p(^3^P°)	^5^G°_3_	G35c				
35	1126.76(5)	88750	1126.75927(5)	0.00	4d^9^_3/2_5s	^2^[3/2]_2_	4d^8^(^3^F)5s5p(^3^P°)	^5^D°_1_	G35c				
84	1125.5403(9)	88846.22	1125.54160(6)	–0.0013	4d^9^_5/2_5s	^2^[5/2]_3_	4d^8^(^3^F)5s5p(^3^P°)	^5^D°_4_	K02c				
64	1122.339(13)	89099.6	1122.34748(6)	–0.008	4d^9^_3/2_5s	^2^[3/2]_1_	4d^9^_5/2_6p	^2^[3/2]°_1_	G35c				
120	1118.9895(9)	89366.34	1118.99053(11)	–0.0010	4d^9^_3/2_5s	^2^[3/2]_2_	4d^8^(^3^F)5s5p(^3^P°)	^5^G°_2_	K02c				
260	1118.5785(9)	89399.18	1118.5793(4)	–0.0008	4d^9^_3/2_5s	^2^[3/2]_1_	4d^8^(^3^F)5s5p(^3^P°)	^5^D°_2_	K02c				
230000	1112.4005(7)	89895.68	1112.4023(7)	–0.0018	4d^10^	^1^S_0_	4d^9^_3/2_5p	^2^[3/2]°_1_	K02c	9.e+08	D+	TW	
50	1111.3619(9)	89979.69	1111.3619(3)	0.0000	4d^9^_5/2_5s	^2^[5/2]_2_	4d^8^(^3^F)5s5p(^3^P°)	^5^D°_3_	K02c				
550000	1106.9930(7)	90334.81	1106.9922(7)	0.0008	4d^10^	^1^S_0_	4d^9^_3/2_5p	^2^[1/2]°_1_	K02c	6.9e+08	D+	B97	
490	1105.3407(7)	90469.84	1105.34083(21)	–0.0001	4d^9^_3/2_5s	^2^[3/2]_2_	4d^9^_3/2_6p	^2^[5/2]°_2_	K02c				
500	1101.5221(7)	90783.47	1101.52218(17)	–0.0001	4d^9^_3/2_5s	^2^[3/2]_2_	4d^9^_3/2_6p	^2^[1/2]°_1_	K02c				
530	1098.242(12)	91054.6	1098.22009(5)	0.022	4d^9^_3/2_5s	^2^[3/2]_1_	4d^8^(^3^F)5s5p(^3^P°)	^5^D°_1_	G35c				
550	1097.3638(7)	91127.48	1097.3641(3)	–0.0003	4d^9^_3/2_5s	^2^[3/2]_2_	4d^9^_3/2_6p	^2^[5/2]°_3_	K02c				
150	1096.7758(7)	91176.34	1096.77568(8)	0.0001	4d^9^_3/2_5s	^2^[3/2]_2_	4d^9^_3/2_6p	^2^[3/2]°_1_	K02c				
550	1095.6237(7)	91272.21	1095.62361(17)	0.0001	4d^9^_5/2_5s	^2^[5/2]_2_	4d^9^_5/2_6p	^2^[7/2]°_3_	K02c				
310	1093.8620(7)	91419.21	1093.86220(12)	–0.0002	4d^9^_3/2_5s	^2^[3/2]_2_	4d^9^_3/2_6p	^2^[3/2]°_2_	K02c				
460	1092.2152(7)	91557.05	1092.2153(3)	–0.0001	4d^9^_5/2_5s	^2^[5/2]_3_	4d^8^(^3^F)5s5p(^3^P°)	^5^D°_3_	K02c				
260	1090.8380(7)	91672.64	1090.83862(11)	–0.0006	4d^9^_3/2_5s	^2^[3/2]_1_	4d^8^(^3^F)5s5p(^3^P°)	^5^G°_2_	K02c				
410	1085.8184(7)	92096.43	1085.81955(6)	–0.0011	4d^9^_5/2_5s	^2^[5/2]_2_	4d^9^_5/2_6p	^2^[3/2]°_1_	K02c				
670	1084.8911(7)	92175.15	1084.89320(5)	–0.0021	4d^9^_5/2_5s	^2^[5/2]_2_	4d^9^_5/2_6p	^2^[5/2]°_2_	K02c				
550	1083.6971(7)	92276.71	1083.69712(4)	0.0000	4d^9^_5/2_5s	^2^[5/2]_2_	4d^9^_5/2_6p	^2^[5/2]°_3_	K02c				
610	1082.2921(7)	92396.50	1082.2923(4)	–0.0002	4d^9^_5/2_5s	^2^[5/2]_2_	4d^8^(^3^F)5s5p(^3^P°)	^5^D°_2_	K02c				
820	1082.1458(7)	92408.99	1082.1457(7)	0.0001	4d^9^_5/2_5s	^2^[5/2]_3_	4d^9^_5/2_6p	^2^[3/2]°_2_	K02c				
110	1080.09(5)	92585	1080.0670(7)	0.02	4d^9^_3/2_5s	^2^[3/2]_2_	4d^8^(^3^F)5s5p(^3^P°)	^3^D°_3_	G35c				
1200	1077.8631(7)	92776.16	1077.86310(20)	0.0000	4d^9^_3/2_5s	^2^[3/2]_1_	4d^9^_3/2_6p	^2^[5/2]°_2_	K02c				
740	1077.0106(7)	92849.60	1077.01091(17)	–0.0003	4d^9^_5/2_5s	^2^[5/2]_3_	4d^9^_5/2_6p	2[7/2]°_3_	K02c				
1700	1072.2519(7)	93261.67	1072.25210(3)	–0.0002	4d^9^_5/2_5s	^2^[5/2]_3_	4d^9^_5/2_6p	^2^[7/2]°_4_	K02c				
1700	1069.7171(7)	93482.66	1069.71694(8)	0.0002	4d^9^_3/2_5s	^2^[3/2]_1_	4d^9^_3/2_6p	^2^[3/2]°_1_	K02c				
1000	1069.2823(7)	93520.67	1069.28243(8)	–0.0001	4d^9^_5/2_5s	^2^[5/2]_3_	4d^8^(^3^F)5s5p(^3^P°)	^5^G°_4_	K02c				
1300	1068.4019(7)	93597.74	1068.40155(8)	0.0003	4d^9^_5/2_5s	^2^[5/2]_2_	4d^8^(^3^F)5s5p(^3^P°)	^5^G°_3_	K02c				
580	1066.9452(7)	93725.53	1066.94527(11)	–0.0001	4d^9^_3/2_5s	^2^[3/2]_1_	4d^9^_3/2_6p	^2^[3/2]°_2_	K02c				
210	1066.6403(7)	93752.32	1066.64026(6)	0.0000	4d^9^_5/2_5s	^2^[5/2]_3_	4d^9^_5/2_6p	^2^[5/2]°_2_	K02c				
2100	1065.4841(7)	93854.05	1065.48406(3)	0.0001	4d^9^_5/2_5s	^2^[5/2]_3_	4d^9^_5/2_6p	^2^[5/2]°_3_	K02c				
1100	1063.224(11)	94053.6	1063.22130(5)	0.002	4d^9^_5/2_5s	^2^[5/2]_2_	4d^8^(^3^F)5s5p(^3^P°)	^5^D°_1_	G35c				
920	1057.6448(7)	94549.70	1057.6457(7)	–0.0009	4d^9^_3/2_5s	^2^[3/2]_2_	4d^8^(^3^F)5s5p(^3^P°)	^3^G°_3_	K02c				
2500	1056.3027(6)	94669.83	1056.30133(10)	0.0014	4d^9^_5/2_5s	^2^[5/2]_2_	4d^8^(^3^F)5s5p(^3^P°)	^5^G°_2_	K02c				
2300	1050.6948(6)	95175.12	1050.69479(8)	0.0000	4d^9^_5/2_5s	^2^[5/2]_3_	4d^8^(^3^F)5s5p(^3^P°)	^5^G°_3_	K02c				
2900	1047.2603(5)	95487.24	1047.2600(6)	0.0004	4d^9^_3/2_5s	^2^[3/2]_1_	4d^8^(^3^F)5s5p(^3^P°)	^3^D°_2_	K02c				
340	1044.1292(8)	95773.59	1044.12986(19)	–0.0007	4d^9^_5/2_5s	^2^[5/2]_2_	4d^9^_3/2_6p	^2^[5/2]°_2_	K02c				
3400	1043.0659(5)	95871.22	1043.0655(5)	0.0004	4d^9^_3/2_5s	^2^[3/2]_2_	4d^8^(^3^F)5s5p(^3^P°)	^3^F°_3_	K02c				
3100	1040.7217(5)	96087.17	1040.72178(15)	–0.0001	4d^9^_5/2_5s	^2^[5/2]_2_	4d^9^_3/2_6p	^2^[1/2]°_1_	K02c				
910	1038.9900(5)	96247.32	1038.99013(10)	–0.0002	4d^9^_5/2_5s	^2^[5/2]_3_	4d^8^(^3^F)5s5p(^3^P°)	^5^G°_2_	K02c				
600	1038.4537(12)	96297.02	1038.4540(6)	–0.0002	4d^9^_3/2_5s	^2^[3/2]_2_	4d^8^(^3^F)5s5p(^3^P°)	^3^D°_1_	K02c				
6300	1037.0110(5)	96430.99	1037.0093(3)	0.0018	4d^9^_5/2_5s	^2^[5/2]_2_	4d^9^_3/2_6p	^2^[5/2]°_3_	K02c				
1600	1036.4837(5)	96480.05	1036.48380(8)	–0.0001	4d^9^_5/2_5s	^2^[5/2]_2_	4d^9^_3/2_6p	^2^[3/2]°_1_	K02c				
8800	1034.2345(5)	96689.87	1034.23469(13)	–0.0002	4d^9^_5/2_5s	^2^[5/2]_2_	4d^8^(^3^F)5s5p(^3^P°)	^5^F°_3_	K02c				
4600	1033.8811(5)	96722.92	1033.88145(11)	–0.0003	4d^9^_5/2_5s	^2^[5/2]_2_	4d^9^_3/2_6p	^2^[3/2]°_2_	K02c				
4500	1031.5741(5)	96939.23	1031.57619(10)	–0.0021	4d^9^_5/2_5s	^2^[5/2]_3_	4d^8^(^3^F)5s5p(^3^P°)	^5^F°_4_	K02c				
6300	1028.5770(5)	97221.70	1028.5769(5)	0.0001	4d^9^_3/2_5s	^2^[3/2]_1_	4d^8^(^3^P)5s5p(^3^P°)	^5^P°_2_	K02c				
14000	1028.3243(5)	97245.59	1028.3239(5)	0.0004	4d^9^_3/2_5s	^2^[3/2]_1_	4d^8^(^3^P)5s5p(^3^P°)	^5^P°_1_	K02c				
12000	1021.5493(5)	97890.53	1021.5491(5)	0.0001	4d^9^_5/2_5s	^2^[5/2]_2_	4d^8^(^3^F)5s5p(^3^P°)	^3^D°_3_	K02c	1.2e+08	D+	TW	
3300	1020.3195(5)	98008.52	1020.31964(25)	–0.0002	4d^9^_5/2_5s	^2^[5/2]_3_	4d^9^_3/2_6p	^2^[5/2]°_3_	K02c				
1900	1017.2918(6)	98300.21	1017.29154(10)	0.0003	4d^9^_5/2_5s	^2^[5/2]_3_	4d^9^_3/2_6p	^2^[3/2]°_2_	K02c				
15000	1015.3869(5)	98484.63	1015.3867(5)	0.0001	4d^9^_5/2_5s	^2^[5/2]_2_	4d^8^(^3^F)5s5p(^3^P°)	^3^D°_2_	K02c	1.5e+08	D+	TW	
27000	1014.1648(5)	98603.30	1014.1646(5)	0.0003	4d^9^_3/2_5s	^2^[3/2]_1_	4d^8^(^3^F)5s5p(^3^P°)	^3^D°_1_	K02c	3.0e+08	D+	TW	
36000	1010.2435(5)	98986.04	1010.24336(7)	0.0001	4d^9^_3/2_5s	^2^[3/2]_2_	4d^8^(^3^F)5s5p(^3^P°)	^1^D°_2_	K02c	6.1e+08	D+	TW	
10000	1008.5859(6)	99148.72	1008.58622(4)	–0.0003	4d^9^_3/2_5s	^2^[3/2]_2_	4d^9^_5/2_4f	^2^[3/2]°_2_	K02c	2.1e+08	D+	TW	
33000	1008.1275(5)	99193.80	1008.12768(5)	–0.0002	4d^9^_3/2_5s	^2^[3/2]_2_	4d^9^_5/2_4f	^2^[3/2]°_1_	K02c				
51000	1007.4503(5)	99260.48	1007.45044(6)	–0.0001	4d^9^_3/2_5s	^2^[3/2]_2_	4d^9^_5/2_4f	^2^[5/2]°_2_	K02c				
7800	1006.4487(6)	99359.26	1006.44894(3)	–0.0002	4d^9^_3/2_5s	^2^[3/2]_2_	4d^9^_5/2_4f	^2^[7/2]°_3_	K02c				
48000	1005.3493(5)	99467.92	1005.3495(5)	–0.0003	4d^9^_5/2_5s	^2^[5/2]_3_	4d^8^(^3^F)5s5p(^3^P°)	^3^D°_3_	K02c	3.8e+08	D+	TW	
42000	1004.9292(5)	99509.50	1004.92924(6)	–0.0001	4d^9^_3/2_5s	^2^[3/2]_2_	4d^8^(^3^F)5s5p(^3^P°)	^1^F°_3_	K02c	1.0e+08	D+	TW	
23000	1001.4694(5)	99853.28	1001.4690(5)	0.0003	4d^9^_5/2_5s	^2^[5/2]_2_	4d^8^(^3^F)5s5p(^3^P°)	^3^G°_3_	K02c				
26000	999.3800(5)	100062.04	999.3805(5)	–0.0005	4d^9^_5/2_5s	^2^[5/2]_3_	4d^8^(^3^F)5s5p(^3^P°)	^3^D°_2_	K02c				
4700	999.2586(10)	100074.20	999.2587(7)	–0.0001	4d^9^_3/2_5s	^2^[3/2]_2_	4d^8^(^1^D)5s5p(^3^P°)	^3^F°_2_	K02c				
42000	997.8142(5)	100219.06	997.8141(5)	0.0001	4d^9^_5/2_5s	^2^[5/2]_2_	4d^8^(^3^P)5s5p(^3^P°)	^5^P°_2_	K02c	3.1e+08	D+	TW	
18000	997.5756(5)	100243.03	997.5760(5)	–0.0004	4d^9^_5/2_5s	^2^[5/2]_2_	4d^8^(^3^P)5s5p(^3^P°)	^5^P°_1_	K02c				
8500	995.9494(6)	100406.71	995.9490(6)	0.0003	4d^9^_5/2_5s	^2^[5/2]_2_	4d^8^(^3^P)5s5p(^3^P°)	^5^P°_3_	K02c				
43000	988.3865(5)	101175.00	988.3869(5)	–0.0005	4d^9^_5/2_5s	^2^[5/2]_2_	4d^8^(^3^F)5s5p(^3^P°)	^3^F°_3_	K02c	2.0e+08	D+	TW	
58000	985.8954(5)	101430.64	985.8952(5)	0.0002	4d^9^_5/2_5s	^2^[5/2]_3_	4d^8^(^3^F)5s5p(^3^P°)	^3^G°_3_	K02c	3.1e+08	D+	TW	
2600	985.2196(17)	101500.21	985.22059(5)	–0.0009	4d^9^_3/2_5s	^2^[3/2]_1_	4d^9^_5/2_4f	^2^[3/2]°_1_	K02c				
14000	984.2449(6)	101600.73	984.2453(6)	–0.0004	4d^9^_5/2_5s	^2^[5/2]_2_	4d^8^(^3^F)5s5p(^3^P°)	^3^D°_1_	K02c				
18000	982.3525(5)	101796.45	982.3528(5)	–0.0003	4d^9^_5/2_5s	^2^[5/2]_3_	4d^8^(^3^P)5s5p(^3^P°)	^5^P°_2_	K02c				
58000	980.5448(5)	101984.12	980.5451(5)	–0.0003	4d^9^_5/2_5s	^2^[5/2]_3_	4d^8^(^3^P)5s5p(^3^P°)	^5^P°_3_	K02c				
76000	976.7483(5)	102380.52	976.7484(5)	0.0000	4d^9^_3/2_5s	^2^[3/2]_1_	4d^8^(^1^D)5s5p(^3^P°)	^3^F°_2_	K02c	2.1e+08	D+	TW	
26000	973.2142(5)	102752.30	973.2142(5)	0.0000	4d^9^_5/2_5s	^2^[5/2]_3_	4d^8^(^3^F)5s5p(^3^P°)	^3^F°_3_	K02c	4.4e+08	D+	TW	
70000	970.8875(5)	102998.54	970.8876(5)	–0.0001	4d^9^_5/2_5s	^2^[5/2]_2_	4d^8^(^3^F)5s5p(^3^P°)	^3^F°_2_	K02c	7.3e+08	D+	TW	
4500	958.8663(9)	104289.83	958.86705(6)	–0.0008	4d^9^_5/2_5s	^2^[5/2]_2_	4d^8^(^3^F)5s5p(^3^P°)	^1^D°_2_	K02c	2.2e+08	D+	TW	
5100	957.3740(8)	104452.39	957.37404(4)	–0.0001	4d^9^_5/2_5s	^2^[5/2]_2_	4d^9^_5/2_4f	^2^[3/2]°_2_	K02c				
18000	956.9606(5)	104497.51	956.96088(5)	–0.0003	4d^9^_5/2_5s	^2^[5/2]_2_	4d^9^_5/2_4f	^2^[3/2]°_1_	K02c				
18000	956.3500(5)	104564.23	956.35062(5)	–0.0006	4d^9^_5/2_5s	^2^[5/2]_2_	4d^9^_5/2_4f	^2^[5/2]°_2_	K02c				
14000	956.2436(5)	104575.86	956.2434(5)	0.0002	4d^9^_5/2_5s	^2^[5/2]_3_	4d^8^(^3^F)5s5p(^3^P°)	^3^F°_2_	K02c	1.5e+08	D+	TW	
35000	954.0780(5)	104813.23	954.07840(5)	–0.0004	4d^9^_5/2_5s	^2^[5/2]_2_	4d^8^(^3^F)5s5p(^3^P°)	^1^F°_3_	K02c				
3500	948.9659(10)	105377.86	948.9657(6)	0.0002	4d^9^_5/2_5s	^2^[5/2]_2_	4d^8^(^1^D)5s5p(^3^P°)	^3^F°_2_	K02c				
11000	943.1323(5)	106029.66	943.13168(4)	0.0006	4d^9^_5/2_5s	^2^[5/2]_3_	4d^9^_5/2_4f	^2^[3/2]°_2_	K02c				
34000	752.793(11)	132838.6	752.7757(3)	0.018	4d^10^	^1^S_0_	4d^9^_5/2_6p	^2^[3/2]°_1_	G35c				
15000	730.816(11)	136833.3	730.8204(4)	–0.004	4d^10^	^1^S_0_	4d^9^_3/2_6p	^2^[1/2]°_1_	G35c				
1600	728.717(11)	137227.4	728.7280(4)	–0.011	4d^10^	^1^S_0_	4d^9^_3/2_6p	^2^[3/2]°_1_	G35c				

a Observed relative intensities from various sources, in terms of total energy flux under the line contour, have been reduced to a common scale corresponding to LTE conditions with effective excitation temperature of 2.9 eV. Variations of sensitivity of registration equipment with wavelength have been removed from intensity values (see text).Where several observations were available, they have been averaged.

b Observed and Ritz wavelengths below 2000 Å are given in vacuum, above that in standard air. Conversion from vacuum to air was made using the five-parameter formula of Peck and Reeder [12]. Uncertainties at a confidence level of one standard deviation are given in parentheses after the value in units of the last decimal place of the value.

c References to observed wavelengths and/or line identifications: B30 – Blair [7]; F95 – Ferrero *et al.* [32]; K02c – Kalus *et al.* [1] with a small calibration correction (see text); G35c, R40ac, R40bc, S28c – Gilbert [2], Rasmussen [3,4], and Shenstone [6], respectively, recalibrated using internal wave number standards based on FTS measurements of Kalus *et al.* [1].

d Accuracy codes for *A*-values are explained in Table 3.

e References to *A*-values: B97 – Biémont *et al.* [11]; C05 – Campos *et al.* [30]; F95c – Ferrero *et al.* [32], renormalized using experimental lifetimes [11]; F95c1 – Ferrero *et al.* [32], recalculated from observed intensities using the Boltzmann equation, Eq. (4); TW – present work.

f Notes: Las – Lasing was observed on these lines in hollow-cathode discharges [21,22]; P – predicted (not observed); S – this line alone determines one of the levels involved in the transition.

**Table 2:** Optimized energy levels of Ag II

Configuration	Term	*J*	Level^a^ (cm^−1^)	Landé *g*	Leading percentages		Alternate designation
4d^10^	^1^S	0	0.00(6)		99		
4d^9^(^2^D_5/2_)5s	^2^[5/2]	3	39168.0177(16)	1.318	100		or 99 ^3^D
4d^9^(^2^D_5/2_)5s	^2^[5/2]	2	40745.3671(13)	1.119	85 + 15 4d^9^(^2^D_3/2_)5s	^2^[3/2]	or 77 ^3^D + 22 ^1^D
4d^9^(^2^D_3/2_)5s	^2^[3/2]	1	43742.7348	0.488	100		or 99 ^3^D
4d^9^(^2^D_3/2_)5s	^2^[3/2]	2	46049.0620(13)	1.028	85 + 14 4d^9^(^2^D_5/2_)5s	^2^[5/2]	or 77 ^1^D + 22 ^3^D
4d^9^(^2^D_5/2_)5p	^2^[3/2]°	2	80176.4601(12)	1.464	92 + 5 4d^9^(^2^D_5/2_)5p	^2^[5/2]°	or 91 ^3^P° + 6 ^3^D°
4d^9^(^2^D_5/2_)5p	^2^[7/2]°	3	82171.7320(13)	1.059	89 + 9 4d^9^(^2^D_5/2_)5p	^2^[5/2]°	or 56 ^3^F° + 36 ^1^F°
4d^9^(^2^D_5/2_)5p	^2^[3/2]°	1	83625.5147	1.410	57 + 42 4d^9^(^2^D_3/2_)5p	^2^1/2]°	or 88 ^3^P° + 5 ^3^D°
4d^9^(^2^D_5/2_)5p	^2^[7/2]°	4	83669.6491(19)	1.249	99		or 99 ^3^F°
4d^9^(^2^D_5/2_)5p	^2^[5/2]°	2	85200.7563(12)	0.851	63 + 31 4d^9^(^2^D_3/2_)5p	^2^[5/2]	or 49 ^3^F° + 23 ^3^D°
4d^9^(^2^D_3/2_)5p	^2^[1/2]°	0	86140.0853(17)		99		or 98 ^3^P°
4d^9^(^2^D_5/2_)5p	^2^[5/2]°	3	86460.6511(13)	1.240	86 + 10 4d^9^(^2^D_5/2_)5p	^2^[7/2]°	or 73 ^3^D° + 24 ^1^F°
4d^9^(^2^D_3/2_)5p	^2^[5/2]°	2	86888.0990(12)	0.889	64 + 31 4d^9^(^2^D_5/2_)5p	^2^[5/2]°	or 46 ^1^D° + 45 ^3^F°
4d^9^(^2^D_3/2_)5p	^2^[5/2]°	3	89134.7240(13)	1.076	96		or 42 ^3^F° + 38 ^1^F°
4d^9^(^2^D_3/2_)5p	^2^[3/2]°	1	89895.5377(12)	1.008	68 + 18 4d^9^(^2^D_3/2_)5p	^2^[1/2]°	or 52 ^1^P° + 46 ^3^D°
4d^9^(^2^D_3/2_)5p	^2^[1/2]°	1	90334.8716(13)	0.52	39 + 30 4d^9^(^2^D_3/2_)5p	^2^[3/2]°	or 47 ^3^D° + 42 ^1^P°
4d^9^(^2^D_3/2_)5p	^2^[3/2]°	2	90887.8423(12)	1.099	92 + 5 4d^9^(^2^D_3/2_)5p	^2^[5/2]°	or 61 ^3^D° + 30 ^1^D°
4d^8^5s^2^	^3^F	4	93932.926(3)		96		
4d^8^5s^2^	^3^F	3	97962.039(6)		97		
4d^8^5s^2^	^3^F	2	99606.031(6)		81 + 14 4d^8^5s^2^	^1^D	
4d^8^5s^2^	^3^P	2	105262.2691(16)		62 + 24 4d^8^5s^2^	^1^D	
4d^8^5s^2^	^3^P	1	108938.4282(17)		96		
4d^8^5s^2^	^3^P	0	109127.691(3)		94		
4d^8^5s^2^	^1^D	2	110773.5186(13)		59 + 32 4d^8^5s^2^	^3^P	
4d^8^5s^2^	^1^G	4	113602.1364(20)		95		
4d^9^(^2^D_5/2_)6s	^2^[5/2]	3	120533.6058(16)	1.293	100		or 100 ^3^D
4d^9^(^2^D_5/2_)6s	^2^[5/2]	2	120911.2048(14)	1.195	99		or 52 ^1^D + 47 ^3^D
4d^9^(^2^D_3/2_)6s	^2^[3/2]	1	125126.4097(14)	0.53	100		or 100 ^3^D
4d^9^(^2^D_3/2_)6s	^2^[3/2]	2	125404.8818(14)	1.11	99		or 52 ^3^D + 47 ^1^D
4d^9^(^2^D_5/2_)5d	2[1/2]	1	125574.8546(24)	1.84	98		or 73 ^3^S + 20 ^3^P
4d^9^(^2^D_5/2_)5d	^2^[9/2	5	126668.189(3)		100		or 99 ^3^G
4d^9^(^2^D_5/2_)5d	^2^[9/2]	4	126679.4151(22)	1.03	99		or 50 ^3^G + 48 ^1^G
4d^9^(^2^D_5/2_)5d	^2^[3/2]	2	126766.4681(19)	1.334	94		or 68 ^3^P + 29 ^3^D
4d^9^(^2^D_5/2_)5d	^2^[3/2]	1	126770.2987(16)	0.90	99		or 50 ^1^P + 26 ^3^D
4d^9^(^2^D_5/2_)5d	^2^[5/2]	3	127211.1186(16)	1.288	96		or 80 ^3^D + 16 ^3^F
4d^9^(^2^D_5/2_)5d	^2^[7/2]	3	127491.0946(18)	0.958	96		or 52 ^1^F + 29 ^3^F
4d^9^(^2^D_5/2_)5d	^2^[5/2]	2	127523.4831(15)	1.006	95		or 51 ^1^D + 23 ^3^D
4d^9^(^2^D_5/2_)5d	^2^[7/2]	4	127608.6762(23)	1.218	99		or 84 ^3^F + 11 ^1^G
4d^8^(^3^F)5s5p(^3^P°)	^5^D°	4	128014.134(5)		85 + 6 4d^8^(^3^F)5s5p(^3^P°)	^5^F°	
4d^9^(^2^D_5/2_)5d	^2^[1/2]	0	128535.1327(24)		77 + 22 4d^9^(^2^D_3/2_)5d	^2^[1/2]	or 85 ^3^P + 14 ^1^S
4d^8^(^3^F)5s5p(^3^P°)	^5^D°	3	130725.061(22)		77 + 8 4d^8^(^3^F)5s5p(^3^P°)	^5^F°	
4d^9^(^2^D_3/2_)5d	^2^[1/2]	1	130762.6969(18)	1.51	98		or 47 ^3^P + 27 ^1^P
4d^9^(^2^D_3/2_)5d	^2^[7/2]	3	131252.5017(22)	0.75?	99		or 84 ^3^G + 10 ^1^F
4d^8^(^3^F)5s5p(^3^P°)	^5^G°	5	131470.919(4)		60 + 31 4d^8^(^3^F)5s5p(^3^P°)	^5^F°	
4d^9^(^2^D_3/2_)5d	^2^[3/2]	1	131507.1120(18)	0.74	99		or 72 ^3^D + 17 ^1^P
4d^9^(^2^D_3/2_)5d	^2^[7/2]	4	131517.079(3)	1.065	99		or 45 ^3^G + 40 ^1^G
4d^9^(^2^D_5/2_)6p	^2^[3/2]°	2	131577.02(7)		92 + 7 4d^9^(^2^D_5/2_)6p	^2^[5/2]°	or 86 ^3^P° + 13 ^3^D°
4d^9^(^2^D_3/2_)5d	^2^[3/2]	2	131790.3202(16)	1.147	98		or 46 ^3^D + 26 ^1^D
4d^9^(^2^D_5/2_)6p	^2^[7/2]°	3	132017.588(14)		87 + 12 4d^9^(^2^D_5/2_)6p	^2^[5/2]°	or 49 ^3^F° + 41 ^1^F°
4d^9^(^2^D_3/2_)5d	^2^[5/2]	2	132155.6138(15)	0.71	98		or 76 ^3^F + 20 ^1^D
4d^9^(^2^D_3/2_)5d	^2^[5/2]	3	132198.3472(21)	1.095	99		or 48 ^3^F + 35 ^1^F
4d^9^(^2^D_5/2_)6p	2[7/2]°	4	132429.668(3)		96		or 96 ^3^F°
4d^8^(^3^F)5s5p(^3^P°)	^5^G°	4	132688.679(7)		61 + 15 4d^8^(^3^F)5s5p(^3^P°)	^5^F°	
4d^9^(^2^D_5/2_)6p	^2^[3/2]°	1	132841.701(5)		96		or 46 ^3^P° + 44 ^1^P°
4d^9^(^2^D_5/2_)6p	^2^[5/2]°	2	132920.339(4)		87 + 6 4d^9^(^2^D_5/2_)6p	^2^[3/2]°	or 53 ^1^D° + 28 ^3^D°
4d^9^(^2^D_5/2_)6p	^2^[5/2]°	3	133022.073(3)		87 + 10 4d^9^(^2^D_5/2_)6p	^2^[7/2]°	or 81 ^3^D° + 18 ^1^F°
4d^8^(^3^F)5s5p(^3^P°)	^5^D°	2	133141.85(3)		76 + 11 4d^8^(^3^P)5s5p(^3^P°)	^5^D°	
4d^8^(^3^F)5s5p(^3^P°)	^5^G°	3	134343.135(7)		74 + 14 4d^8^(^3^F)5s5p(^3^P°)	^5^F°	
4d^9^(^2^D_3/2_)5d	^2^[1/2]	0	134455.9504(21)		76 + 22 4d^9^(^2^D_5/2_)5d	^2^[1/2]	or 78 ^1^S + 14 ^3^P
4d^8^(^3^F)5s5p(^3^P°)	^5^D°	1	134799.164(4)		80 + 14 4d^8^(^3^P)5s5p(^3^P°)	^5^D°	
4d^8^(^3^F)5s5p(^3^P°)	^5^G°	2	135415.323(9)		82 + 8 4d^8^(^3^F)5s5p(^3^P°)	^5^F°	
4d^8^(^3^F)5s5p(^3^P°)	^5^F°	4	136107.052(9)		63 + 11 4d^8^(^3^F)5s5p(^3^P°)	^3^G°	
4d^9^(^2^D_3/2_)6p	^2^[1/2]°	0	136169.811(22)?		99		or 99 ^3^P°
4d^9^(^2^D_3/2_)6p	^2^[5/2]°	2	136518.895(17)		94 + 5 4d^9^(^2^D_3/2_)6p	^2^[3/2]°	or 85 ^3^F° + 14 ^1^D°
4d^9^(^2^D_3/2_)6p	^2^[1/2]°	1	136832.527(14)		93		or 54 ^1^P° + 42 ^3^P°
4d^9^(^2^D_3/2_)6p	^2^[5/2]°	3	137176.52(3)		89		or 45 ^3^F° + 38 ^1^F°
4d^9^(^2^D_3/2_)6p	^2^[3/2]°	1	137225.409(7)		84 + 5 4d^8^5s5p		or 77 ^3^D° + 11 ^3^P°
4d^8^(^3^F)5s5p(^3^P°)	^5^F°	3	137435.220(12)		53 + 15 4d^8^(^3^F)5s5p(^3^P°)	^5^G°	
4d^9^(^2^D_3/2_)6p	^2^[3/2]°	2	137468.255(10)		79		or 48 ^3^D° + 27 ^1^D°
4d^8^(^3^F)5s5p(^3^P°)	^3^D°	3	138635.91(6)		48 + 17 4d^8^(^3^F)5s5p(^3^P°)	^3^F°	
4d^8^(^3^F)5s5p(^3^P°)	^3^D°	2	139230.01(6)		25 + 17 4d^8^(^3^F)5s5p(^3^P°)	^5^F°	
4d^8^(^3^F)5s5p(^3^P°)	^3^G°	3	140598.68(6)		51 + 22 4d^8^(^3^F)5s5p(^3^P°)	^3^D°	
4d^8^(^3^P)5s5p(^3^P°)	^5^P°	2	140964.44(6)		58 + 18 4d^8^(^3^F)5s5p(^3^P°)	^3^D°	
4d^8^(^3^P)5s5p(^3^P°)	^5^P°	1	140988.36(6)		50 + 16 4d^8^(^3^F)5s5p(^3^P°)	^5^F°	
4d^8^(^3^P)5s5p(^3^P°)	^5^P°	3	141152.11(6)		62 + 21 4d^8^(^3^F)5s5p(^3^P°)	^3^G°	
4d^8^(^3^F)5s5p(^3^P°)	^3^F°	3	141920.32(6)		33 + 29 4d^8^(^3^F)5s5p(^3^P°)	^1^F°	
4d^8^(^3^F)5s5p(^3^P°)	^3^D°	1	142346.06(6)		36 + 40 4d^8^(^3^P)5s5p(^3^P°)	^5^P°	
4d^8^(^3^F)5s5p(^3^P°)	^3^F°	2	143743.90(6)		55 + 19 4d^8^(^3^F)5s5p(^3^P°)	^3^D°	
4d^9^(^2^D_5/2_)7s	^2^[5/2]	3	144478.44(9)		100		or 100 ^3^D
4d^9^(^2^D_5/2_)7s	^2^[5/2]	2	144624.16(7)		100		or 57 ^1^D + 43 ^3^D
4d^9^(^2^D_5/2_)4f	^2^[1/2]°	1	145014.040(4)		82 + 17 4d^9^(^2^D_5/2_)4f	^2^[3/2]°	or 80 ^3^P° + 13 ^3^D°
4d^8^(^3^F)5s5p(^3^P°)	^1^D°	2	145035.112(6)		34 + 13 4d^9^4f	^3^P°	
4d^9^(^2^D_5/2_)4f	^2^[11/2]°	6	145181.205(4)		100		or 99 ^3^H°
4d^9^(^2^D_5/2_)4f	^2^[11/2]°	5	145182.054(4)		100		or 55 ^1^H° + 45 ^3^H°
4d^9^(^2^D_5/2_)4f	2[3/2]°	2	145197.750(4)		70 + 14 4d^9^(^2^D_5/2_)4f	^2^[5/2]°	or 36 ^3^D° + 28 ^3^P°
4d^9^(^2^D_5/2_)4f	^2^[3/2]°	1	145242.847(5)		83 + 16 4d^9^(^2^D_5/2_)4f	^2^[1/2]°	or 51 ^1^P° + 47 ^3^D°
4d^9^(^2^D_5/2_)4f	^2^[5/2]°	2	145309.528(6)		74 + 9 4d^9^(^2^D_5/2_)4f	^2^[3/2]°	or 42 ^1^D° + 32 ^3^F°
4d^9^(^2^D)4f	^2^[9/2]°	5	145389.480(3)		99		or 86 ^3^G° + 8 ^3^H°
4d^9^(^2^D_5/2_)4f	^2^[7/2]°	3	145408.300(3)		93		or 40 ^3^F° + 29 ^1^F°
4d^9^(^2^D_5/2_)4f	^2^[7/2]°	4	145427.113(3)		50 + 49 4d^9^(^2^D_5/2_)4f	^2^[9/2]°	or 47 ^3^G° + 39 ^3^F°
4d^9^(^2^D_5/2_)4f	^2^[9/2]°	4	145497.860(3)		49 + 48 4d^9^(^2^D_5/2_)4f	^2^[7/2]°	or 51 ^1^G° + 34 ^3^F°
4d^8^(^3^F)5s5p(^3^P°)	^1^F°	3	145558.556(6)		27 + 13 4d^9^4f	^3^D°	
4d^8^(^1^D)5s5p(^3^P°)	^3^F°	2	146123.25(6)		46 + 12 4d^8^(^3^P)5s5p(^3^P°)	^5^D°	
4d^9^(^2^D_3/2_)7s	^2^[3/2]	1	149082.86(9)		100		or 99 ^3^D
4d^9^(^2^D_3/2_)7s	^2^[3/2]	2	149182.18(8)		100		or 57 ^3^D + 42 ^1^D
4d^9^(^2^D_5/2_)8s	^2^[5/2]	3	155088.95(23)		100		or 100 ^3^D
4d^9^(^2^D_5/2_)8s	^2^[5/2]	2	155161.42(22)		100		or 59 ^1^D + 41 ^3^D
4d^9^(^2^D_3/2_)8s	^2^[3/2]	1	159695.21(24)		100		or 100 ^3^D
4d^9^(^2^D_3/2_)8s	^2^[3/2]	2	159740.36(24)		100		or 58 ^3^D + 41 ^1^D
Ag III (4d^9 2^D_5/2_)	Limit		173283(7)				
Ag III (4d^9 2^D_3/2_)	Limit		177892(7)				

a Uncertainties of separations from the 4d^9^(^2^D_5/2_)5p ^2^[3/2]_1_ level are given in parentheses after the value in units of the last decimal place of the value. To obtain the uncertainties of the excitation energies, they must be combined in quadrature with the uncertainty of the ground level, ±0.06 cm^−1^. All uncertainties are given on the level of one standard deviation.

**Table 3:** Transition probability uncertainty code

Symbol	Uncertainty in *A*-value	Uncertainty in log(*gf*)
A	≤ 3 %	≤ 0.013
B+	≤ 7 %	≤ 0.03
B	≤ 10 %	≤ 0.04
C+	≤ 18 %	≤ 0.08
C	≤ 25 %	≤ 0.11
D+	≤ 40 %	≤ 0.18
D	≤ 50 %	≤ 0.24
E	> 50 %	> 0.24
